# Uncovering genetic mechanisms of kidney aging through transcriptomics, genomics, and epigenomics

**DOI:** 10.1016/j.kint.2018.10.029

**Published:** 2019-03

**Authors:** Joshua Rowland, Artur Akbarov, James Eales, Xiaoguang Xu, John P. Dormer, Hui Guo, Matthew Denniff, Xiao Jiang, Parisa Ranjzad, Alicja Nazgiewicz, Priscilla Ribeiro Prestes, Andrzej Antczak, Monika Szulinska, Ingrid A. Wise, Ewa Zukowska-Szczechowska, Pawel Bogdanski, Adrian S. Woolf, Nilesh J. Samani, Fadi J. Charchar, Maciej Tomaszewski

**Affiliations:** 1 Division of Cardiovascular Sciences, School of Medical Sciences, Faculty of Medicine, Biology and Health, University of Manchester, Manchester, UK; 2 Department of Cellular Pathology, University Hospitals of Leicester, Leicester, UK; 3 Division of Population Health, Health Services Research and Primary Care, Faculty of Medicine, Biology and Health, University of Manchester, Manchester, UK; 4 Department of Cardiovascular Sciences, University of Leicester, Leicester, UK; 5 Division of Cell Matrix Biology and Regenerative Medicine, School of Biological Sciences, Faculty of Biology Medicine and Health, University of Manchester, Manchester, UK; 6 Faculty of Health and Life Sciences, Federation University Australia, Ballarat, Victoria, Australia; 7 Department of Urology and Uro-oncology, Karol Marcinkowski University of Medical Sciences, Poznan, Poland; 8 Department of Treatment of Obesity, Metabolic Disorders and Clinical Dietetics, Poznan University of Medical Sciences, Poznan, Poland; 9 Department of Health Care, Silesian Medical College, Katowice, Poland; 10 Department of Internal Medicine, Diabetology and Nephrology, Medical University of Silesia, Zabrze, Poland; 11 Department of Paediatric Nephrology, Royal Manchester Children’s Hospital, Manchester University National Health Service Foundation Trust, Manchester Academic Health Science Centre, Manchester, UK; 12 Leicester National Institute for Health Research Biomedical Research Centre, Glenfield Hospital, Leicester, UK; 13 Department of Physiology, University of Melbourne, Parkville, Victoria, Australia; 14 Division of Medicine and Manchester Heart Centre, Manchester University National Health Service Foundation Trust, Manchester Academic Health Science Centre, Manchester, UK

**Keywords:** aging, epigenome, genetics, kidney, transcriptome

## Abstract

Nephrons scar and involute during aging, increasing the risk of chronic kidney disease. Little is known, however, about genetic mechanisms of kidney aging. We sought to define the signatures of age on the renal transcriptome using 563 human kidneys. The initial discovery analysis of 260 kidney transcriptomes from the TRANScriptome of renaL humAn TissuE Study (TRANSLATE) and the Cancer Genome Atlas identified 37 age-associated genes. For 19 of those genes, the association with age was replicated in 303 kidney transcriptomes from the Nephroseq resource. Surveying 42 nonrenal tissues from the Genotype–Tissue Expression project revealed that, for approximately a fifth of the replicated genes, the association with age was kidney-specific. Seventy-three percent of the replicated genes were associated with functional or histological parameters of age-related decline in kidney health, including glomerular filtration rate, glomerulosclerosis, interstitial fibrosis, tubular atrophy, and arterial narrowing. Common genetic variants in four of the age-related genes, namely *LYG1*, *PPP1R3C*, *LTF* and *TSPYL5*, correlated with the trajectory of age-related changes in their renal expression. Integrative analysis of genomic, epigenomic, and transcriptomic information revealed that the observed age-related decline in renal TSPYL5 expression was determined both genetically and epigenetically. Thus, this study revealed robust molecular signatures of the aging kidney and new regulatory mechanisms of age-related change in the kidney transcriptome.

see commentary on page 492

Aging is associated with a decline in health and integrity of the human kidney.^1^ Indeed, from the fourth decade of life, the glomerular filtration rate falls by 8 ml/min per 1.73 m^2^ every 10 years.^2^ Moreover, the kidney undergoes structural remodeling with age.^3^, ^4^ Macroscopically, kidney mass and blood flow decline with age.^3^, ^5^, ^6^ These changes are likely to contribute to the known increase in risk of chronic kidney disease (CKD) with age.^7^

Genetic mechanisms may impact renal aging, and previous gene expression profiling experiments highlighted molecules associated with the development of age-related changes in the kidney.^8^, ^9^ These studies had several caveats: they used small numbers of samples, typically fewer than 100; lacked robust replication in independent cohorts; and used microarrays to quantify the expression of genes in the kidney. In contrast to microarrays, next-generation RNA-sequencing (RNA-seq) allows for an unbiased hypothesis-free approach to transcriptome profiling and increased sensitivity to detect changes in genes with low levels of expression such as long noncoding RNAs.^10^, ^11^ Thus, previous studies may not have defined certain genes relevant to kidney aging because of limitations in technology or sample size, whereas the genes that were identified were not replicated in other populations.

It also is unclear whether the expression of age-associated kidney genes is controlled, at least in part, by genetically and/or epigenetically inherited variations in DNA. Single differences in DNA sequences determine phenotypic variation in health and disease, for example, through differential effects on gene expression.^12^, ^13^ Several recent studies have reported associations between common single-nucleotide polymorphisms (SNPs) and human aging,^14^ as well as renal gene expression.^15^ Epigenetic modifications, such as DNA methylation, also can modify gene expression.^16^ Indeed, aging is accompanied by genome-wide hypomethylation and site-specific hypermethylation, at least in nonrenal tissues.^17^ The age-related changes in regional 5′—C—phosphate—G—3′ site (CpG) methylation across tissues have been used to derive a global epigenetic signature as a measure of biological age.^18^, ^19^ Nevertheless, patterns of DNA methylation are highly variable across different tissues and a majority of methylation studies have been conducted on tissues other than the kidney.^20^

In this study, we identified robust signatures of age on the renal transcriptome by studying a large assembly of human kidneys characterized by RNA-seq. We identified several genes that showed similar age-related changes across different human tissues, while others represented kidney-specific aging signatures. We also found associations between these signatures and certain functional and structural measures of kidney health. Finally, by integrating genomic, epigenomic, and transcriptomic information, we uncovered new regulatory mechanisms underlying the expression of age-related signatures in the human kidney.

## Results

### Signatures of age on kidney transcriptome: discovery analysis

**Figure 1:**
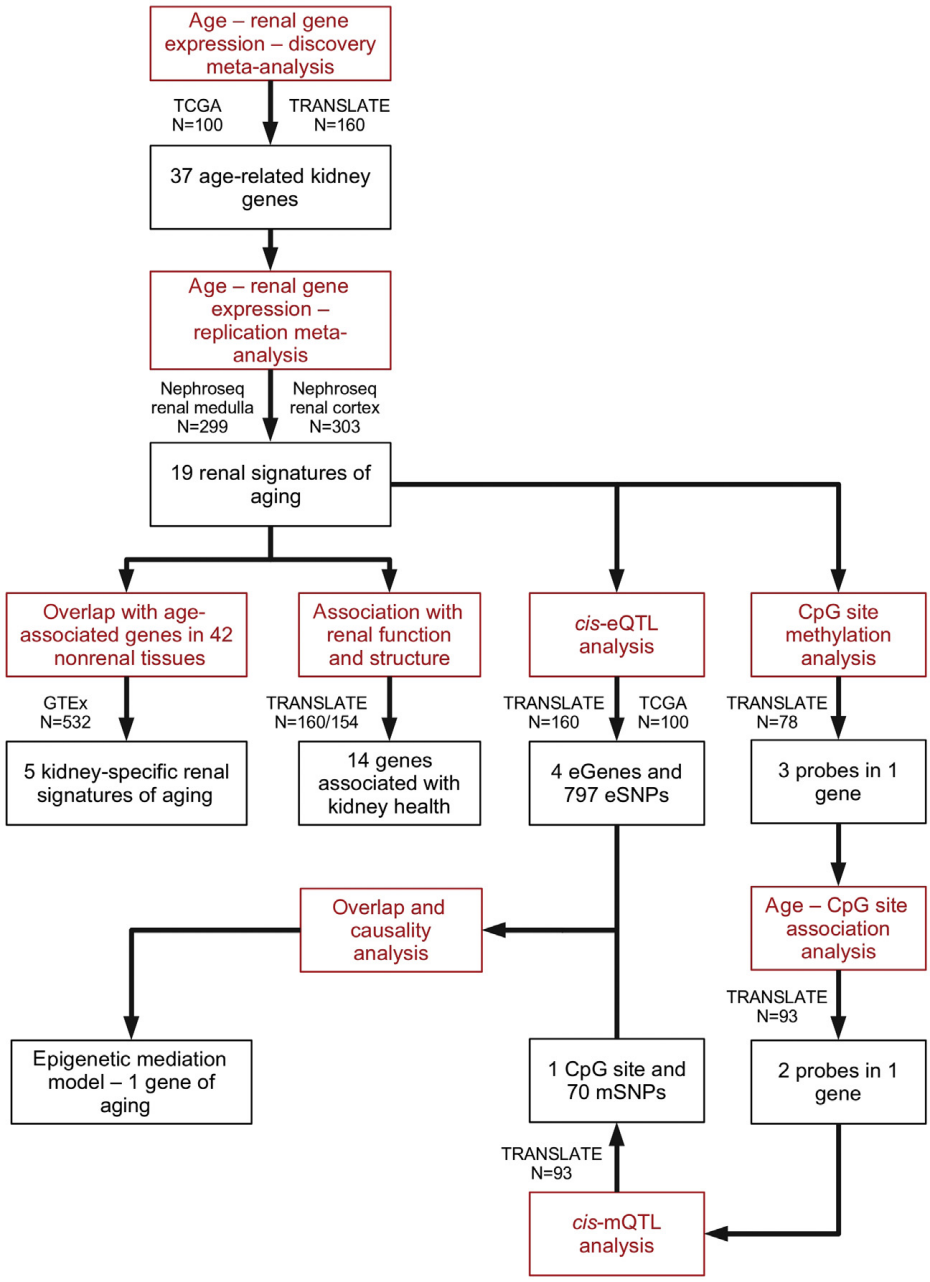
**Illustration of key stages of the project together with the main outcomes.** TCGA, The Cancer Genome Atlas; TRANSLATE Study, TRANScriptome of renaL humAn TissuE Study; *Nephroseq* renal cortex, a combination of 3 separate studies of renal cortex/glomerulus from *Nephroseq* resource; *Nephroseq* renal medulla, a combination of 3 separate studies of renal medulla/tubulointerstitium from *Nephroseq* resource; GTEx, Genotype–Tissue Expression project; *cis*-eQTL, *cis*-expression quantitative trait locus; *cis*-mQTL, *cis*-methylation quantitative trait locus; eGene, gene whose renal expression is associated with at least 1 single-nucleotide polymorphism in-*cis* after correction for multiple testing; mSNP, gene whose renal DNA methylation is associated with at least 1 single-nucleotide polymorphism in-*cis* after correction for multiple testing; CPG, 5’—C—phosphate—G—3’ site; eSNP, transcriptionally active single-nucleotide polymorphism.

**Figure 2:**
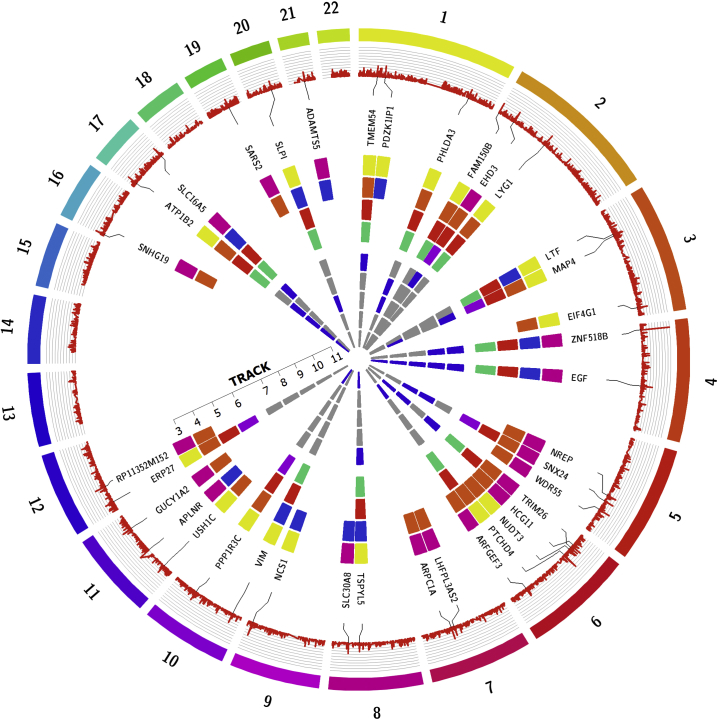
**Overview of kidney genes associated with age.** Chromosome numbers appear at the top of the circle and proceed clockwise. Track 1: The level of statistical significance (-log_10_*P* values) from meta-analysis of association between renal gene expression and age in the TRANScriptome of renaL humAn TissuE (TRANSLATE) Study and The Cancer Genome Atlas (TCGA). Track 2: Symbols of 37 kidney genes associated with age in the meta-analysis of the TRANSLATE Study and TCGA after the correction for multiple testing. Track 3: Direction of association between expression of a gene and age (based on β-coefficients from the meta-analysis of the TRANSLATE Study and TCGA). Yellow, positive: gene is upregulated with increasing age; pink, negative: gene is downregulated with increasing age. Track 4: Prior evidence for association with aging determined via search of PubMed, GenAge, and AgeMap databases. Blue, genes previously associated with age; orange, no previous association with age. Track 5: Kidney genes associated with age in *Nephroseq* resource (red). Track 6: Kidney-specific (in purple) and ubiquitous (in green) signatures of age (based on the analysis of nonrenal tissues). Tracks 7 to 11: Associations between replicated age-related kidney genes and estimated glomerular filtration rate (track 7), glomerulosclerosis (track 8), interstitial fibrosis (track 9), tubular atrophy (track 10), and arterial/arteriolar narrowing (track 11). At least nominally significant associations are shown in blue.

The design and delivery of the project is shown in the flow chart in Figure 1. In total, 160 patients from the TRANScriptome of renaL humAn TissuE (TRANSLATE) Study and 100 individuals from The Cancer Genome Atlas (TCGA) were included in the discovery analysis. The clinical characteristics of both studies are shown in Table 1. A total of 15,791 kidney genes common to both data sets were included in the analysis. After correction for multiple testing, 37 genes were associated with age (Figure 2, Supplementary Figure S1, and Supplementary Table S1). Of these, 33 were protein-coding and 4 were long noncoding RNAs. Biological characteristics of these genes are shown in Supplementary Table S2. Only 1 gene, *EGF*, overlapped with 307 genes implicated in aging from the GenAge database.^21^ Taking advantage of clinical information available in the TRANSLATE Study, we further examined the potential effects of common cofounders or comorbidities on the association between age and renal gene expression. The sensitivity analysis showed that after adjusting for body mass index, hypertension, and diabetes mellitus, 30 of the 37 genes (81%) retained associations with age in the TRANSLATE Study at the nominal level of statistical significance of 5% (Supplementary Table S3).

**Table 1:** Demographic and selected clinical characteristics of the TRANSLATE Study and TCGA

Characteristics	TRANSLATE Study	TCGA
N	160	100
Age, yr	63.2 ± 10.4	61.4 ± 13.3
Age range, yr	30–87	28–86
Male sex	99 (62%)	69 (69%)
White–European ethnicity	160 (100%)	100 (100%)
Body mass index, kg/m^2^	28.2 ± 5.2	–
Hypertension	116 (73%)	–
Diabetes	29 (18%)	
eGFR, ml/min per 1.73 m^2^	75.3 ± 19.1	–
Glomerular sclerosis	59/82/13/0	–
Interstitial fibrosis	70/70/14/0	–
Tubular atrophy	67/69/17/1	–
Arterial/arteriolar narrowing	15/94/39/6	–

eGFR, estimated glomerular filtration rate; TCGA, The Cancer Genome Atlas; TRANSLATE, TRANScriptome of renaL humAn TissuE Study.

Data are counts (and percentages where appropriate), means and SDs or ranges. For histologic phenotypes (n = 154), data are numbers with Remuzzi’s scale from 0 to 3 (0/1/2/3), where 0 indicates none to minimal damage and 3 indicates maximal damage.

### Signatures of age on kidney transcriptome: replication analysis

We identified a total of 303 glomerular/renal cortex transcriptomes and 299 tubulointerstitial/renal medulla transcriptomes from eligible studies in *Nephroseq*.^22^ Of 37 age-associated genes, 32 were available for replication. Separate meta-analyses of renal cortex and medulla showed 12 and 18 associations with age, respectively, after correction for multiple testing (Supplementary Tables S4 and S5). A total of 19 kidney genes (59.3% of those available for this analysis) associated with age in the discovery analysis replicated in the same direction of association in *Nephroseq* (Figure 2). Additional sensitivity analysis showed that most (60%) of the age-associated genes that replicated exclusively in apparently healthy kidney tissues^8^ overlapped with those identified in the replication limited to samples from patients with kidney disease.^23^, ^24^

### Ubiquitous and kidney-specific gene signatures of aging

Three (16%) of the replicated genes, *EGF*, *EDH3*, and *LYG1*, showed enriched kidney RNA/protein expression in the Human Protein Atlas^25^ (Supplementary Table S2). Although most of the age-associated kidney genes were abundant in other tissues, we reasoned that age-related changes in expression of these genes may be different compared with nonrenal tissues. To identify genes correlating with age exclusively in the kidney, we first conducted an association analysis between age and gene expression in 42 nonrenal Genotype–Tissue Expression (GTEx) project tissues from 532 individuals. The overview of the tissues included in this analysis and the demographic characteristics of the GTEx subjects are shown in Supplementary Tables S6 and S7. This analysis showed between 0 and 276 associations of age with gene expression in different tissues (Supplementary Table S6). All of our 19 replicated kidney genes were available for an overlap analysis with the transcriptomic signatures of age in the GTEx project (Supplementary Table S8). Under the initially selected statistical criteria, 15 (79%) of these showed no overlap with age-associated GTEx genes in this analysis (Figure 2 and Supplementary Table S8). Further sensitivity analyses were conducted under several alternative scenarios, with a different number of probabilistic estimations of expression residuals–derived factors in the nonrenal GTEx tissues. These showed that only 5 genes, comprising 26% of the replicated genes, showed no association with age in any of the statistical scenarios in any nonrenal tissues (Supplementary Table S9). As such, these genes, *EDH3*, *ERP27*, *MAP4*, *PPP1R3C*, and *SNX24*, represent tissue-specific signatures of age on the kidney transcriptome.

### Robust gene signatures of kidney aging and their correlation with functional and structural dimensions of kidney health

Of 19 replicated genes, 14 showed at least 1 nominally significant association with 1 of 5 biochemical or histologic measures of renal functional and structural integrity known to deteriorate with age (i.e., estimated glomerular filtration rate, glomerulosclerosis, tubular atrophy, interstitial fibrosis, and arterial/arteriolar narrowing) in up to 160 individuals from the TRANSLATE Study (Supplementary Tables S10–S14, Figure 2, and Supplementary Figure S2). The direction of these associations was always consistent with the expected age-related decline in kidney health. After correction for multiple testing, 10 genes retained their statistically significant associations with at least 1 measure of kidney health (Supplementary Tables S10–S14 and Supplementary Figure S2), as follows: *ATP1B2*, *EGF*, *EHD3*, *MAP4*, *SLPI*, *TMEM54*, *SLC16A5*, *ZNF518B*, *TRIM26*, and *TSPYL5*. Statistically, the most significant relationship was detected between decreasing kidney expression of *TSPYL5* and increasing arterial/arteriolar narrowing (*P* = 6.3 × 10^-5^, false discovery rate, 0.0015) (Supplementary Table S14). Further sensitivity analyses showed that age adjustment reduced or completely abolished the statistical significance of all genes (Supplementary Tables S10–S14), consistent with a key role of aging in the uncovered associations between renal gene expression and indices of kidney health.

### Cross-species validation of age-related human kidney genes in rodents

Of 19 genes showing association with human age at the renal expression level, 8 had murine homologs (*Egf*, *Ltf*, *Lyg1*, *Phlda3*, *Slc16a5*, *Slpi*, *Snx24*, and *Vim*) profiled on the gene expression microarray/available for *in silico* replication.^26^ The renal expression of 4 of these genes (*Egf*, *Phlda3*, *Slpi*, and *Vim*) showed a statistically significant association with murine age, at least at the nominal level of statistical significance (Supplementary Table S15). *Phlda3*, *Slpi*, and *Vim* also were associated with the histologic measure of age-related decline in kidney health, namely glomerular membrane thickening and tubular degeneration, in mice (Supplementary Table S15). In the absence of availability of *TSPYL5* for validation in mice, we measured age-related changes in the expression of this gene in rat kidneys.^27^ This analysis showed reduced renal expression of *TSPYL5* with age (Supplementary Figure S3). Collectively, these studies show a high level, approximately 50%, of cross-species replication of age-related kidney genes between man and rodents.

### Immunohistochemistry of TSPYL5 in human kidneys

**Figure 3:**
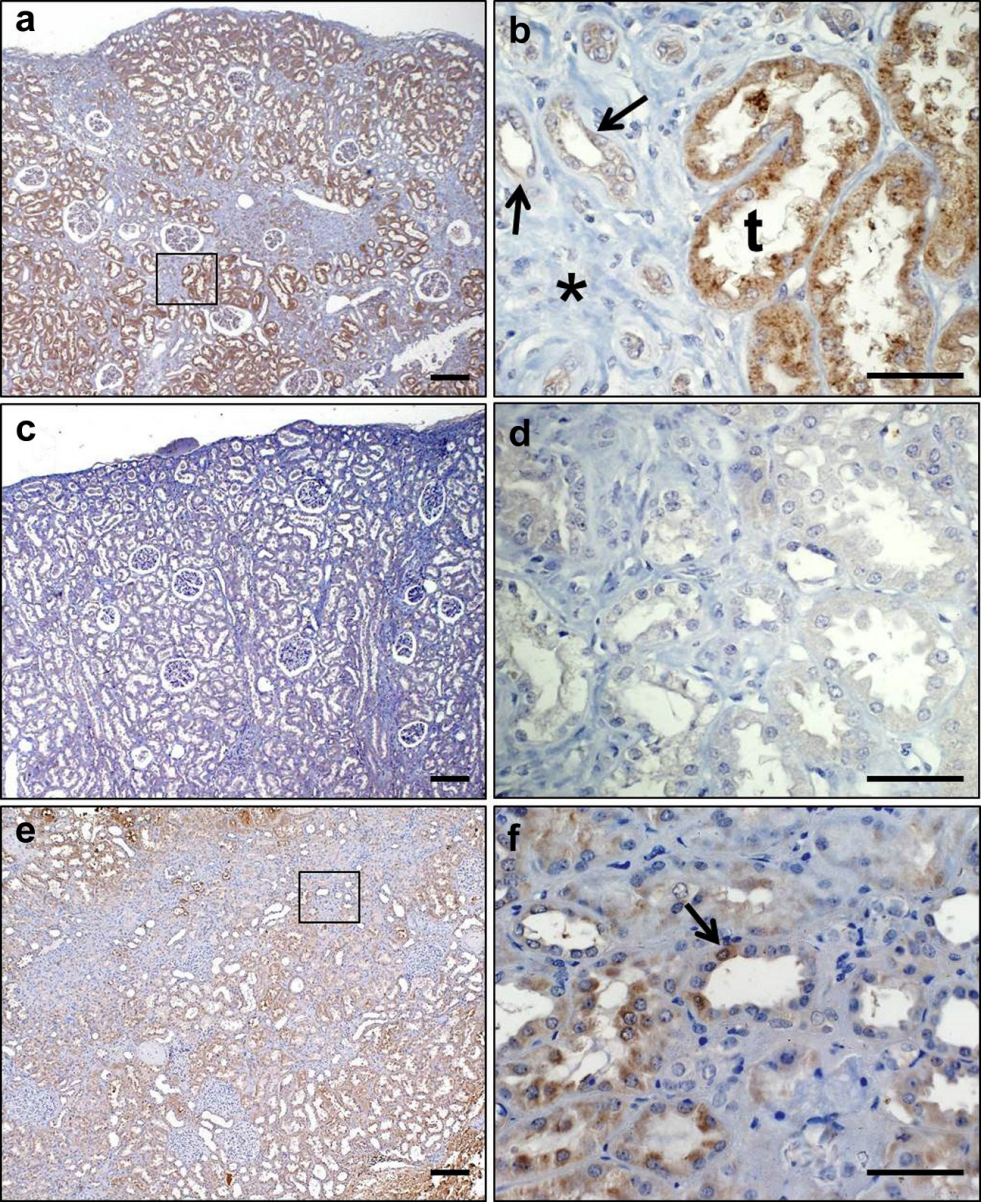
**Immunohistochemistry of TSPYL5 in human kidneys.** (**a,b,e,f**) Sections were immunostained for TSPYL5 (brown signal), (**c,d**) except where the primary antibody was omitted. All sections were counterstained with hematoxylin (blue nuclei). (**a**) Low-power overview of kidney cortex of a 58-year-old individual showing glomeruli and tubules. (**b**) High-power view of boxed area in panel **a**. Larger tubules (t) have prominent TSPYL5 immunostaining, with a cytoplasmic pattern. Smaller tubules (arrows) in the fibrotic area (*) appear to have less prominent TSPYL5 signals. (**c,d**) Negative controls in which goat anti-rabbit secondary antibody was applied but the primary antibody was omitted. (**e**) Low-power overview of kidney cortex of an 81-year-old individual showing prominent zones of atrophic tubules and fibrosis. (**f**) High-power view from sample in panel **e**: in some tubules TSPYL5 signals appear confined to a subset of cells (arrow). Bars = (**a,c,e**) 200 μm, and (**b,d,f**) 50 μm. To optimize viewing of this image, please see the online version of this article at www.kidney-international.org.

In kidneys from 9 TRANSLATE Study individuals, immunostaining for TSPYL5 detected a common pattern of signals in tubules, in a cytoplasmic location, with 2 representative samples shown in Figure 3. Further quantitative analysis of TSPYL5 immunostaining showed numerically lower signal intensity in kidneys from older individuals (age, >60 yr) when compared with those younger than 60 years, but the difference was not statistically significant (*P* = 0.176) (Supplementary Figure S4).

### Genetic regulation of renal expression of age-associated genes

To determine the extent of genotype-dependent variation in the expression of the 19 age-associated genes, we performed in-*cis* meta-expression quantitative trait locus analysis of the TRANSLATE Study and TCGA data with a window of 1 Mb. After correction for multiple testing, 4 (21%) genes, *LTF*, *LYG1*, *PPP1R3C*, and *TSPYL5*, showed significant correlations with 797 variants in*-cis* (Table 2 and Supplementary Tables S16 and S17). All most significant variants (best transcriptionally active single-nucleotide variants–best eSNPs) mapped to noncoding regions of the genome; 3 of them within actively transcribed chromatin regions in adult kidney tissue (Supplementary Table S18). Genotypes of the best eSNPs showed fairly constant effects on the age-related changes in renal gene expression (Supplementary Figure S5). We further examined associations between the genotypes of 797 *PPP1R3C*, *TSPYL5*, *LYG1*, and *LTF* eSNPs and estimated the glomerular filtration rate in 110,527 individuals from the CKDGen Consortium.^28^ Of 723 variants available for analysis, 105, 55, and 4 eSNPs in *PPP1R3C*, *TSPYL5*, and *LYG1*, respectively, showed an association with estimated glomerular filtration rate in the CKDGen Consortium, at least at the nominal level of statistical significance (Supplementary Table S19). The best eSNPs of *PPP1R3C* (rs7077656) and *TSPYL5* (rs2567772) were among the eSNPs associated with an estimated glomerular filtration rate in the CKDGen Consortium. Thus, many genetic variants that control expression changes of kidney aging genes also are associated with a surrogate of kidney function.

**Table 2:** Association between age-related kidney genes and their best eSNPs: *cis*-expression quantitative locus meta-analysis of the TRANSLATE Study and TCGA

Chr	Gene symbol	rsID	Distance from gene to SNP, base pairs	Alleles	β	SE	*P* value	Permutation *P* value	FDR
10	*PPP1R3C*	rs7077656	–18,450	A/G	–0.428	0.062	5.44E-12	5.00E-04	1.64E-03
8	*TSPYL5*	rs2567772	–4622	G/A	0.287	0.043	1.58E-11	5.00E-04	1.64E-03
3	*LTF*	rs6763280	0	G/A	0.283	0.043	4.48E-11	5.00E-04	1.64E-03
2	*LYG1*	rs6734290	20,339	G/T	–0.244	0.040	1.46E-09	5.00E-04	1.64E-03

Alleles, reference/alternate allele; β, coefficient from linear regression models; Chr, chromosome; FDR, false discovery rate; permutation *P* value, *P* value obtained based on 2000 permutations; rsID, reference SNP ID; SNP, single-nucleotide polymorphism.

### DNA methylation of age-associated genes and their expression

**Figure 4:**
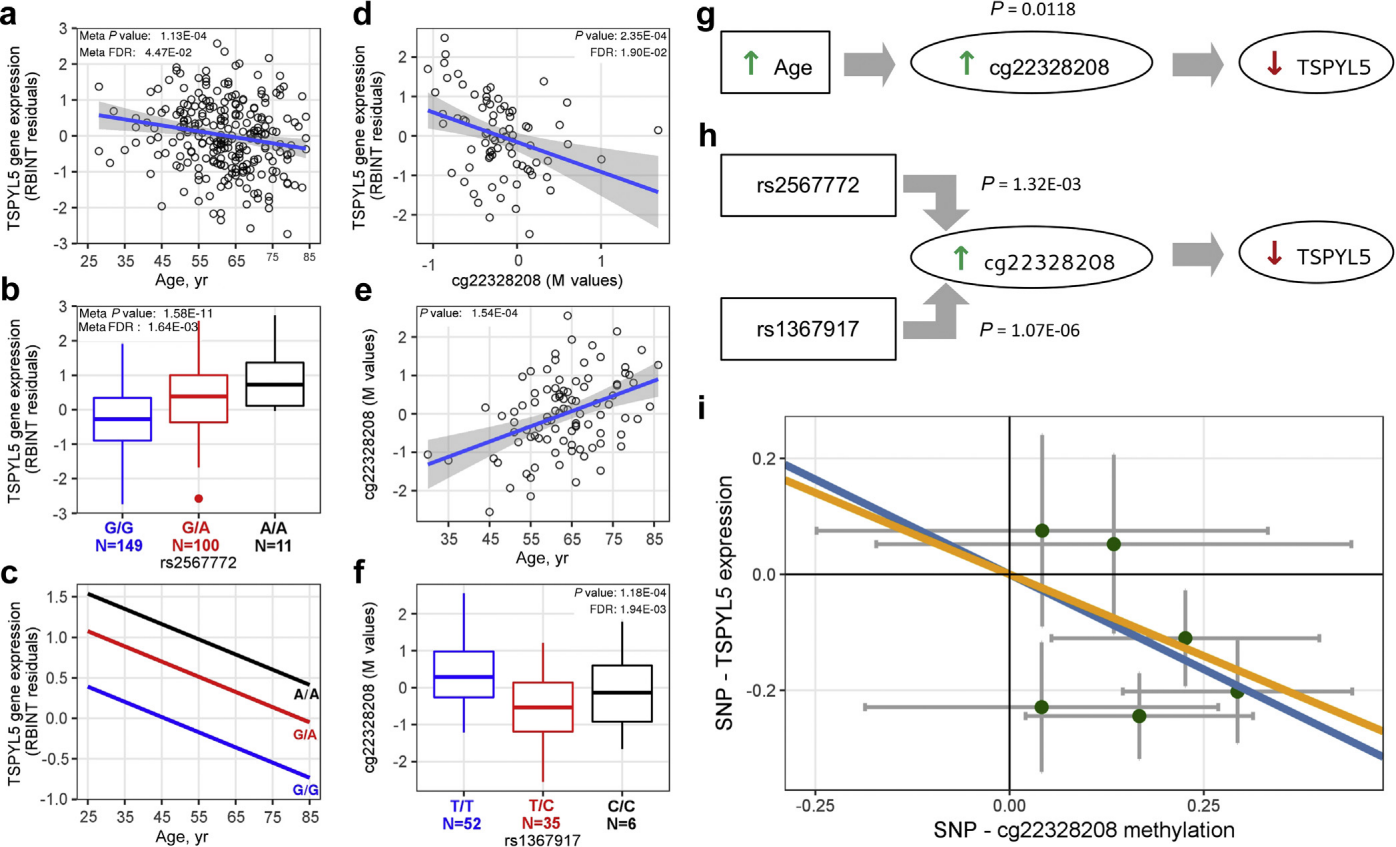
***TSPYL5:* associations between renal expression, methylation, age, and genotype.** (**a**) Association between kidney expression of *TSPYL5* and age in the meta-analysis of the TRANScriptome of renaL humAn TissuE (TRANSLATE) Study and The Cancer Genome Atlas (TCGA) study. Meta *P* value, level of statistical significance from the joint analysis; meta false discovery rate (FDR), the level of statistical significance after the correction for multiple testing. (**b**) Association between kidney expression of *TSPYL5* and its best eSNP (rs256772) in the meta-analysis of TRANSLATE and TCGA studies. Meta *P* value, level of statistical significance from the meta-analysis; meta FDR, the level of statistical significance after the correction for multiple testing. (**c**) Trajectories of age-related reduction in kidney expression of *TSPYL5* stratified on the genotype of rs256772 in the combined TRANSLATE and TCGA studies. (**d**) Association between kidney expression of *TSPYL5* and the extent of renal DNA methylation within the cg22328208 probe in the TRANSLATE Study. *P* value, nominal level of statistical significance; FDR, the level of statistical significance after correction for multiple testing. (**e**) Association between the extent of renal DNA methylation within the cg22328208 probe and age in the TRANSLATE Study. *P* value, level of statistical significance. (**f**) Association between the extent of renal DNA methylation within the cg22328208 probe and its best single-nucleotide variant with effect on methylation (mSNP) (rs1367917). *P* value, nominal level of statistical significance. (**g**) Effect of age on the kidney expression of *TSPYL5* is mediated through the extent of renal DNA methylation within cg22328208 in the TRANSLATE Study. (**h**) Effects of the best eSNP (rs256772) and the best mSNP (rs1367917) on the renal expression of TSPYL5 are mediated through the extent of renal DNA methylation within cg22328208 in the TRANSLATE Study. (**i**) The causal effect of methylation at cg22328208 on kidney expression of *TSPYL5* using 6 independent genetic instruments (transcriptionally active single-nucleotide polymorphisms [SNPs]) in Mendelian randomization by inverse variance weighting (orange line) and weighted median (blue line). The green dots with arrow bars represent genetic associations with kidney expression of *TSPYL5* against genetic associations with cg22328208 methylation (with 95% confidence intervals). RBINT, rank-based inverse normal transformation.

To investigate possible effects of DNA methylation on gene expression, we examined whether differences in CpG methylation of kidney DNA in proximity to 19 age-associated genes correlate with their renal expression in 78 TRANSLATE Study individuals. We examined the extent of DNA methylation in all CpG sites mapping to 1000 base pairs from either side of the age-associated gene. After correction for multiple testing, methylation of 1 gene, *TSPYL5*, showed an inverse association with its renal expression; the strongest association was identified for cg22328208 probe mapping to the *TSPYL5* promoter (*P* = 2.3×10^-4^, Permutation P [*P*_perm_] = 1 × 10^-3^, false discovery rate, 0.019) (Table 3, Supplementary Table S20, and Figure 4). We further confirmed that methylation of the *TSPYL5* promoter was associated with a reduction in its expression using data generated in *in vitro* experiments (Supplementary Table S21).

**Table 3:** Associations between *TSPYL5* CpG methylation, *TSYL5* renal expression, and chronological age in the TRANSLATE Study

Chr	CpG probe	Trait	β	SE	FDR
8	cg22328208	*TSPYL5* expression	–0.545	0.135	0.019
		Age	0.016	0.004	<0.001

β, coefficient from linear regression models; Chr, chromosome; CpG probe, DNA methylation probe; FDR, false discovery rate.

### Association between age, renal methylation, and expression of *TSPYL5*

We detected the association between age and 3 CpG probes associated with the expression of *TSPYL5* in 93 TRANSLATE Study individuals (Supplementary Table S22). The positive association of cg22328208 methylation with age is in agreement with the demonstrated negative association between renal expression of *TSPYL5* and a decrease in the kidney abundance of *TSPYL5* with age (Table 3, Supplementary Table S22, and Figure 4). We then confirmed the association between cg22328208 methylation and age in an independent collection of 126 kidney samples from TCGA (Supplementary Table S23). Further mediation analysis in the TRANSLATE Study showed that the effect of age on cg22328208 is primary to changes in *TSPYL5* expression in the kidney (*P* = 0.0118) (Figure 4). Collectively, these data show that age-related increase in methylation of the *TSPYL5* promoter in the kidney translates into a decrease in the renal expression of *TSPYL5*.

### Genotype-dependent effect on kidney DNA methylation of *TSPYL5*

Genetic variation is known as one of the regulatory mechanisms for CpG methylation in human tissues. Therefore, we sought to identify common variants associated with methylation of cg22328208 in 93 TRANSLATE Study individuals. This *cis-*methylation quantitative trait locus analysis used a window of 33,172 base pairs, the maximum distance between *TSPYL5* and its eSNPs plus its own length, from the target CpG site and showed that after correction for multiple testing, cg22328208 was associated with 70 SNPs (Supplementary Table S24). The strongest association was identified for rs1367917 (Figure 4). These data confirmed that interindividual differences in renal DNA methylation of *TSPYL5* are dependent on genotype.

### Mediation analysis and causality between kidney methylation and expression of *TSPYL5*

We jointly investigated the identified *cis*-expression quantitative trait locus and *cis*-methylation quantitative trait locus of *TSPYL5* in 93 TRANSLATE Study samples that had informative eQTL and mQTL data: 62 of 65 eSNPs overlapped with the set of 70 single-nucleotide variants with effect on methylation (mSNPs) (Supplementary Table S25). The mediation analysis based on the set of overlapping SNPs, *TSPYL5* methylation, and expression was consistent with the epigenetic mediation model: both the best mSNP (rs1367917; *P* = 1.07 × 10^-6^) and the best eSNP (rs2567772; *P* = 1.32 × 10^-3^) were linked to *TSPYL5* expression through cg22328208 methylation (Figure 4). Further Mendelian randomization using *TSPYL5* SNPs as instruments, cg22328208 methylation as exposure, and *TSPYL5* expression as outcome, and both inverse variance weighting and weighted median methods showed a causal effect of cg22328208 methylation on renal *TSPYL5* expression; the effect estimates were -0.563 (95% confidence interval, -0.326 to -0.801) and -0.655 (95% confidence interval, -0.071 to -1.24) (Figure 4). The Mendelian randomization–Egger regression indicated that there was no pleiotropy of the instruments (*P* = 0.291).

### Functional analysis of *TSPYL5* locus in silico

We used Roadmap Epigenomics chromatin immunoprecipitation sequencing data for 4 key histone modifications to determine chromatin state segmentation in an adult kidney and overlapped chromatin states and chromatin immunoprecipitation-sequencing signal intensity across the *TSPYL5* locus (Supplementary Figure S6). The cg22328208 CpG site overlapped with the transcription start site of the *TSPYL5* gene and is in a transcription start site chromatin region (1_TssA) in adult kidney tissue.^29^ It is part of a CpG island. Histone modification intensity in the region showed a strong enrichment for H3K4me3 (a mark of high specificity to promoter regions). The best eSNP (rs2567772) and mSNP (rs1367917) are 9.1 kb and 23.5 kb downstream from *TSPYL5* and map to a weakly transcribed chromatin state (5_TxWk) and quiescent chromatin (15_Quies) in adult human kidney, respectively (Supplementary Table S26). Integrative analysis of high-resolution (5–10 kb) genome-wide high throughput chromosome confirmation capture chromatin interaction data^30^ showed statistically significant physical interactions between regions proximal to the mSNP and eSNP (or their proxies) and the promoter region of *TSPYL5* (Supplementary Figure S6 and Supplementary Table S27).

## Discussion

Our study provides several insights into age-related gene expression changes within the kidney. First, we identify genes whose expression not only robustly correlates with age in independent data sets but also is associated with biochemical/histologic measures of age-related changes in renal health. Second, we provide evidence for the key role of common genetic variants as determinants of lifelong, age-related changes in the renal signatures of aging. Finally, through integration of information from the genome, epigenome, and transcriptome we identify *TSPYL5* as a robust gene whose age-related change in kidney expression is mediated through genetically determined hypermethylation of its promoter.

Only 6 (31.6%) (*EGF*, *LTF*, *SLPI*, *SLC16A5*, *VIM*, and *TSPYL5*) of 19 robust signatures of age-related changes in kidney transcriptome were linked to human aging or age-related kidney diseases in previous studies. For example, *EGF* expression is known to correlate inversely with CKD, renal fibrosis, and rapidly progressive glomerulonephritis.^23^, ^31^ Changes in renal expression of 2 other genes (*LTF* and *SLPI*) were linked to acute kidney injury.^32^, ^33^ *LTF* is an antibacterial, anti-inflammatory, and antioxidant factor providing an innate line of defense against a host of injury stimuli.^34^ *SLPI* protects epithelial cells from the effects of inflammation and the activity of endogenous proteolytic enzymes.^35^, ^36^ To this end, the identified increase in the expression of *LTF* and *SLP1* with age may represent a kidney repair mechanism in response to activation of aging-related processes in that organ. The degree of overlap between age-related genes and those that constitute the transcriptomic signatures of kidney injury identified before suggests the existence of a commonality in transcriptional pathways of renal damage repair irrespective of the initial insult.

Our data link a majority of the replicated age-associated kidney genes to the functional and structural parameters of renal health known to deteriorate with age. Indeed, the age-related decline in glomerular filtration rate affects up to 57% to 85% of individuals in the general population.^37^, ^38^, ^39^ Glomerular sclerosis, interstitial fibrosis, tubular atrophy, and arterial narrowing represent the age-related changes in the structural microarchitecture of the kidney.^39^ Their gradual progression inevitably leads to a loss of functional nephron reserve and atrophy, increasing the susceptibility of elderly patients to severe presentations of CKD and other renal disorders.^3^ The associations between these phenotypes and our age-related genes suggest that a majority of the uncovered signatures are not silent bystanders but more likely are contributors to the key processes underpinning a gradual functional and structural involution of renal tissue with aging.

We also show a powerful effect of the inherited variation in DNA sequence on the kidney expression of genes related to aging. Our data suggest that for approximately 1 in 5 renal genes robustly associated with age, common variants mapping to their proximity determine the lifelong pattern of their expression in the kidney. This means that the pace with which kidneys age is determined at least in part at birth and those who inherited a genotype promoting steeper expression changes of aging signatures may be at higher risk of age-related decline in kidney health (given the demonstrated overlap between age-related changes in renal gene expression and markers of functional and structural kidney damage). Thus, prediction of the magnitude of age-related changes in transcriptome based on genotype could lead to the development of new important diagnostic avenues. Indeed, noninvasive genotyping of faster renal aging risk variants might be an attractive strategy for early screening of age-related kidney diseases and suitability of organ donation in kidney transplantation.^4^

One of the most interesting genes uncovered by our study is *TSPYL5*, a member of the nucleosome assembly protein superfamily known for their role in transcriptional regulation and cell cycle.^40^ *TSPYL5* was implicated in the processes operating at the intersection of cellular senescence and carcinogenesis.^40^ Specifically, *TSPYL5* was suggested to impact on p53 expression and the activity of telomerase,^41^, ^42^ and was predicted to associate with telomere length.^43^ The recent *in vitro* TSPYL5 knockdown experiments in human pluripotent stem cells showed that silencing of this gene leads to changes in the expression of numerous pathways, including those responsible for changes in DNA conformation, epigenetics, and telomere organization.^44^ These processes map onto the basic molecular and cellular underpinnings of aging including regulation of chromatin architecture, epigenetic control of transcription, and cellular senescence.^45^

Our results show that an age-related decrease in the renal expression of *TSPYL5* is most likely mediated directly by CpG methylation, with the strongest signal mapping to the 5’ region of the gene. Jung *et al.*^46^ determined that this segment of *TSPYL5* including exon 1 contains the transcription start site and lies within a CpG island. This was supported by our analysis *in silico*. The association between the methylation and expression of *TSPYL5* has been reported previously in other human tissues including both apparently healthy and diseased tissues.^47^, ^48^ The methylation of *TSPYL5* also was associated with other age-related conditions including Huntington’s disease.^49^ Notably, the degree of hypermethylation of *TSPYL5* promoter in the kidney remains under genetic control and is linked causally to the renal expression of this gene, but the strongest mSNP is located more than 20,000 base pairs from the *TSPYL5* promoter. Similar intronic/intergenic SNPs distant to the methylated CpG sites were shown as the regulators of their methylation in previous studies.^47^ Our *in silico* studies further suggest that long-range chromatin interactions between the chromatin segments overlapping with the *TSPYL5* promoter and the distant regions where the best mSNP maps are plausible explanations for the detected associations.

Certainly, there were limitations of our analyses. The noncancerous renal tissues analyzed in both the TRANSLATE and TCGA cohorts were sourced from nephrectomies performed for primary kidney cancers. However, our previous investigations showed that the presence of cancer does not affect the transcriptome of kidney tissue collected from the cancer-unaffected part of the kidney.^50^ Moreover, the tissues within the replication resource that validated the associations between 19 renal genes and age came mostly from noncancer patients. Thus, it is unlikely that the history of renal cancer in the donors had a confounding effect on our results. We also appreciate the limited size of our kidney methylation data set in the TRANSLATE Study. The power limitation was the most likely reason why our study uncovered only 1 independent association between age-related gene expression and methylation after the stringent correction for multiple testing. However, 63% of our renal signatures showed nominally significant associations with kidney DNA methylation (Supplementary Table S20) and/or were correlated with CpG methylation in other human tissues.^47^, ^51^ Finally, we acknowledge the limitations of the quantitative analysis of TSPYL5 immunostaining restricted to 9 human kidneys. These samples represent only a small fraction of the set of renal tissues from more than 500 individuals examined at the transcriptome level. Thus, the arising results likely were underpowered for defining changes in the target protein with age. In the future, the complete spectrum of protein changes will need to be measured in several hundreds of samples using high-throughput proteomic approaches.

Despite these caveats, our analysis linked age-related changes in kidney expression to genome and epigenome and show compelling evidence for promoter hypermethylation as the causal mechanism for an age-related decrease in renal expression of one of the uncovered signatures.

We stress that our discovery analysis was based exclusively on RNA-seq, the state-of-the-art method of RNA profiling to measure transcript abundance. We also applied stringent statistical methods in the analyses of association between gene signatures and aging. Previous studies relying on much smaller numbers of kidney samples, collected largely in a similar manner to the TRANSLATE Study and TCGA but profiled using microarrays, used less-stringent statistical thresholds and lacked an independent replication. Therefore, it is not possible to exclude the role of a type 1 error in relation to approximately 1000 reported associations with age in these investigations.^8^ Most notably, the magnitude of discovery for age-associated kidney genes in our analysis is consistent with that for a majority of nonrenal GTEx tissues processed by RNA-seq and examined using similar statistical pipelines. Therefore, we are confident that the identified genes represent robust signatures of age on kidney transcriptome.

In summary, with 260 kidneys examined at the discovery stage and more than 300 kidneys analyzed in the replication phase, our study was a large analysis of changes in the human renal transcriptome in relation to aging. We uncovered new robust gene signatures of kidney aging, showed their relevance to age-related decline in renal health, and demonstrated the potential of integrative *-omics* to uncover new regulatory mechanisms underpinning age-related changes in renal gene expression.

## Methods

Discovery analysis of association between gene expression and chronological age was conducted using data from 2 projects: the TRANSLATE Study^50^, ^52^ and TCGA.^53^ First, separate analyses were performed on the 2 data sets, then the results were combined using an inverse-variance method. Kidney transcriptome profiling in both projects was conducted by RNA-seq. Replication analysis was conducted using data from *Nephroseq*, a platform of comprehensive renal disease gene expression data sets.^22^ Within *Nephroseq*, we performed an analysis of age-associated genes using a collection of apparently healthy renal tissue from Rodwell *et al.*,^8^ and 2 data sets based on specimens from patients with kidney disease by Ju *et al.*^23^ and Sampson *et al.*,^24^ separately in kidney cortex and medulla. The number of age-associated genes in 42 nonrenal tissues and the kidney specificity of replicated genes was examined using the data from the GTEx project.^54^, ^55^ Replicated genes were examined further for association with renal function and structure, age-related renal changes in rodents and cell lines, common genotypes (expression quantitative trait locus analysis), associations with CKD, and kidney DNA methylation. Kidney tissues immunostaining was performed using 2 antibodies raised against *TSPYL5*. Each antibody provided similar patterns on kidney cortex sections from 9 TRANSLATE Study individuals, spanning an age range of 30 to 81 years. The relevant DNA methylation sites were examined for association with chronological age and genotype (methylation quantitative trait locus analysis).

Gene expression was quantified in transcripts per million and normalized using logarithmic transformation, quantile normalization, and rank-based inverse normal transformation in both the TRANSLATE Study and TCGA. The extent of technical variation in the normalized gene expression data was determined using the probabilistic estimation of the expression residual method.^56^ All association analyses were conducted using multiple linear regression controlling for sex, the top 3 genotype principal components, and technical factors if appropriate. The statistical significance was determined based on the false discovery rate either using the Benjamini–Hochberg method or the method of Storey *et al.*^57^, ^58^ We performed causal inference and mediation analysis and Mendelian randomization to explore the causal drivers of changes in gene expression. Bioinformatic analyses consisted of regulatory characterization of age-associated genes (Roadmap Epigenomics chromatin state), examining their organ specificity (Human Protein Atlas^25^) and long-range chromatin interactions (high throughput chromosome confirmation capture data^59^). Further details regarding the methods are provided in the Supplementary Data.

## Disclosure

All the authors declared no competing interests.

## Supplementary Material

Supplementary Data

Figure S1 Analysis of association between age and kidney gene expression, a meta-analysis of the TRANScriptome of renaL humAn TissuE (TRANSLATE) Study and The Cancer Genome Atlas (TCGA). β, a coefficient of regression from the meta-analysis, is plotted on the X axis, statistical significance [-log_10_ (*P* value)] is plotted on the Y axis (inversely log-transformed for ease of interpretation), the dotted line shows a threshold for correction for multiple testing [false discovery rate (FDR), 5%], genes upregulated with age at FDR <0.05 are shown in green, genes down-regulated with age at FDR < 0.05 are shown in red.

Figure S2 Analysis of association between replicated age-associated genes and 5 measures of renal health in the TRANScriptome of renaL humAn TissuE (TRANSLATE) Study. Different colors show the magnitude of statistical significance for association between the expression of each individual gene and a given phenotype. Row 1: Genes whose expression increased with age in the TRANSLATE Study/The Cancer Genome Atlas (TCGA) analysis are shown in dark green. Genes whose expression decreased with age in the TRANSLATE Study/TCGA analysis are shown in red. Rows 2 to 6: Genes whose expression is associated positively with the given renal phenotype after correction for multiple testing [false discovery rate (FDR), < 5%] are shown in dark green. Genes whose expression is associated positively with the given renal phenotype at the nominal level (*P* < 5%) are shown in light green. Genes whose expression is associated inversely with the given renal phenotype after correction for multiple testing (FDR, <5%) are shown in red. Genes whose expression is associated inversely with the given renal phenotype at the nominal level (*P* < 5%) are shown in orange. Genes whose expression was not associated significantly with the given renal phenotype are shown in gray. The estimated glomerular filtration rate (eGFR) was calculated based on the EPI-CKD formula.

Figure S3 Association of renal expression of *TSPYL5* with age in rats. *P* value: level of statistical significance from analysis of variance (ANOVA).

Figure S4 Analysis of the difference in immunohistochemistry-derived signal intensity for kidney *TSPYL5* between younger (age, ≤60 yr) and older (age, >60 yr) individuals from the TRANScriptome of renaL humAn TissuE (TRANSLATE) Study. N, number of individuals; *P* value, level of statistical significance using the Mann–Whitney *U* test.

Figure S5 Associations between age, renal expression of signature genes, and their best eSNPs in the TRANScriptome of renaL humAn TissuE (TRANSLATE) Study and The Cancer Genome Atlas (TCGA). (**A**) Associations between the renal expression of each gene and age in the meta-analysis of the TRANSLATE Study and TCGA. Meta *P* value, level of statistical significance from the meta-analysis of both studies; meta false discovery rate (FDR), the level of statistical significance after correction for multiple testing. (**B**) Renal expression of each gene stratified on the genotype of the best eSNP in the meta-analysis of the TRANSLATE Study and TCGA. meta *P* value, level of statistical significance from the meta-analysis of both studies; meta FDR, the level of statistical significance after correction for multiple testing. (**C**) Trajectories of age-related changes in renal expression of the 4 genes stratified on the genotype of the best respective eSNP in the meta-analysis of the TRANSLATE Study and TCGA.

Figure S6 *TSPYL5* functional annotation to the locus on chromosome 8. Hi-C chromatin interactions are shown as gray arcs, the intensity of gray is determined by the number of times the interaction was observed. The best mSNP and eSNP are shown as large points in blue and yellow, respectively. mSNP and eSNP statistical proxies (r^2^ > 0.8 in 1000 Genomes European individuals) are shown as smaller points in blue and yellow, respectively. The CpG site (cg22328208) is shown in dark purple and its parent CpG island (chromosome 8: 98289605-98290404; 25% CpG content) is shown in light purple. The *TSPYL5* gene is shown as a gray region, with the coding exon black. Chromatin state information from adult kidney tissue is shown below the gene, red denotes transcription start site regions, yellow indicates enhancer regions, and green indicates transcribed regions. Input histone modification chromatin immunoprecipitation (ChIP)-seq data signal is shown at the bottom; H3K4me3 is shown in red, H3K4me1 is shown in yellow, and H3K36me3 in green. The histone modification signal is calculated as the Loess smoothed density of ChIP-seq reads across the region, colors are from Roadmap Epigenomics.

Table S1 Meta-analysis of association between renal genes and age in the TRANSLATE Study and TCGA.

Table S2 Functional characterization of genes associated with kidney aging in the TRANSLATE Study and TCGA.

Table S3 Effect of adjustment for comorbidities (body mass index, hypertension, and diabetes) on association between age and renal expression of 37 genes from the discovery analysis–sensitivity analysis in the TRANSLATE Study.

Table S4 Replication of associations between renal genes and age in the renal cortex/glomerular compartment: meta-analysis of 3 separate studies from *Nephroseq* resource.

Table S5 Replication of associations between renal genes and age in the renal medulla/tubulointerstitial compartment: meta-analysis of 3 separate studies from *Nephroseq* resource.

Table S6 GTEx tissues included in the analysis of association between age and gene expression.

Table S7 Demographic characteristics of individuals from GTEx.

Table S8 Analysis of association between age and expression of age-related renal genes in nonrenal GTEx tissues.

Table S9 Sensitivity analyses of association between age and expression of age-related renal genes in nonrenal GTEx tissues.

Table S10 Analysis of association between 19 robust age-related genes and estimated glomerular filtration rate in the TRANSLATE Study.

Table S11 Analysis of association between 19 robust age-related genes and glomerular sclerosis in the TRANSLATE Study.

Table S12 Analysis of association between 19 robust age-related genes and interstitial fibrosis in the TRANSLATE Study.

Table S13 Analysis of association between 19 robust age-related genes and tubular atrophy in the TRANSLATE Study.

Table S14 Analysis of association between 19 robust age-related genes and arterial/arteriolar narrowing in the TRANSLATE Study.

Table S15 Cross-species validation of 19 genes showing age-related changes in kidney expression: analysis of murine model of renal aging.

Table S16 Gene signatures of kidney aging and their best eSNPs: meta analysis of the TRANSLATE Study and TCGA.

Table S17 Gene signatures of kidney aging and their eSNPs: meta-analysis of the TRANSLATE Study and TCGA.

Table S18 Functional annotations of the 4 best eSNPs associated with renal expression of age-related gene signatures.

Table S19 Association of eSNPs (for PPP1R3C, TSPYL5, LYG1, and LTF) with estimated glomerular filtration rate in CKDGen genome-wide association study.

Table S20 CpG sites associated with renal expression of age-related genes in the TRANSLATE Study.

Table S21 The effect of *TSPYL5* methylation on its expression: cell lines.

Table S22 Association between methylation and age for 3 probes associated with renal expression of *TSPYL5* in the TRANSLATE Study.

Table S23 Analysis of association between age and the extent of kidney DNA methylation at the *TSPYL5* promoter: replication in TCGA study.

Table S24 Association between age- and gene-expression–related CpG sites and genetic variants in-*cis*: all significant mSNPs from mQTL analysis in the TRANSLATE Study (eQTL best eSNP is highlighted).

Table S25 Overlap between mSNPs and eSNPs for *TSPYL5*.

Table S26 Functional annotation of the best mSNP for cg22328208 in the TRANSLATE Study.

Table S27 Chromatin interaction data from the 4DGenome database used in Figure S6.

Table S28 Postimputation quality control for eQTL analysis in the TRANSLATE Study and TCGA.

Table S29 Postimputation quality control for mQTL analysis in the TRANSLATE Study.
