# Supplementary material for: Uncovering genetic mechanisms of kidney aging through transcriptomics, genomics, and epigenomics
Source: Kidney Int. 2019 Mar;95(3):624–35. doi: 10.1016/j.kint.2018.10.029 (PMC6390171; doi:10.1016/j.kint.2018.10.029)
Supplement: Supplementary Data [file mmc1.docx]

Supplement

**Table of Contents**

[TRANSLATE Study – recruitment, sample processing and phenotypes 3](#_Toc527325181)

[TCGA – recruitment, sample processing and phenotypes 4](#_Toc527325182)

[RNA extraction and RNA-seq analyses in TRANSLATE Study and TCGA 4](#_Toc527325183)

[RNA-seq quality control 5](#_Toc527325184)

[RNA-seq normalisation 6](#_Toc527325185)

[DNA analyses in TRANSLATE Study and TCGA 6](#_Toc527325186)

[Genotype quality control 7](#_Toc527325187)

[Genotype principal components 7](#_Toc527325188)

[Genotype imputation 8](#_Toc527325189)

[DNA methylation 8](#_Toc527325190)

[*Nephroseq* – kidney gene expression profiles 9](#_Toc527325191)

[GTEx project – recruitment, sample processing and phenotypes 10](#_Toc527325192)

[TSPYL5 – human kidney immunohistochemistry 10](#_Toc527325193)

[Statistical and bioinformatic analyses 11](#_Toc527325194)

[Discovery analysis 11](#_Toc527325195)

[Replication analysis 12](#_Toc527325196)

[GTEx non-renal tissue analysis 12](#_Toc527325197)

[Renal function and structure analysis 13](#_Toc527325198)

[Cross-species validation of age-related human kidney genes in mice 13](#_Toc527325199)

[Association between renal expression of TSPYL5 and age in rats 13](#_Toc527325200)

[cis-eQTL meta-analysis 14](#_Toc527325201)

[Association between age-related eSNPs and estimated glomerular filtration rate – CKDGen Consortium 15](#_Toc527325202)

[DNA methylation and gene expression analysis 15](#_Toc527325203)

[DNA methylation and age analysis 16](#_Toc527325204)

[mQTL analysis 16](#_Toc527325205)

[Causality analysis 17](#_Toc527325206)

[Bioinformatic analysis 17](#_Toc527325207)

[Figure S1. Analysis of association between age and kidney gene expression – meta-analysis of TRANSLATE Study and TCGA. 19](#_Toc527325208)

[Figure S2. Analysis of association between replicated age-associated genes and 5 measures of renal health in TRANSLATE Study. 20](#_Toc527325209)

[Figure S3. Association of renal expression of TSPYL5 with age in rats. 21](#_Toc527325210)

[Figure S4. Analysis of the difference in immunohistochemistry-derived signal intensity for kidney TSPYL5 between younger (aged 60 years or younger – <60) and older (aged over 60 years – >60) individuals from TRANSLATE Study. 22](#_Toc527325211)

[Figure S5: Associations between age, renal expression of signature genes and their best eSNPs in TRANSLATE Study and TCGA. 23](#_Toc527325212)

[Figure S6. TSPYL5: functional annotation to the locus on chromosome 8. 24](#_Toc527325213)

[References 25](#_Toc527325214)

# TRANSLATE Study – recruitment, sample processing and phenotypes

We utilised data collected in TRANScriptome of renaL humAn TissuE (TRANSLATE) Study.^S1,S2^ In brief, the study recruited individuals of white European ancestry with no personal history of primary nephropathy and with eligibility for unilateral elective nephrectomy because of sporadic (non-hereditary) non-invasive renal cancer. Specimens were taken from the healthy (unaffected by cancer) pole of the kidney immediately after surgery and immersed in RNAlater^S1,S2^ for further molecular profiling. An additional kidney sample was secured for histology/DNA extraction and a serum sample for standard biochemistry. Demographic and clinical phenotypes were collected for each individual.^S1–3^ We extracted relevant demographics (age, sex), clinical (BMI, hypertension and diabetes) and both biochemical (serum creatinine) and histological (glomerulosclerosis, tubular atrophy, interstitial fibrosis and arterial/arteriolar narrowing) phenotypes from the TRANSLATE Study database. In brief, hypertension was defined as reported previously.^S2^ Diabetes was diagnosed based on either self-declaration of diabetes and/or remaining on hypoglycaemic medications. Circulating concentrations of creatinine were measured by modified kinetic Jaffe method (calibrated to an IDMS reference measurement procedure) on Advia Chemistry 1200 (Siemens) and used in calculating estimated glomerular filtration rate (eGFR) with CKD-EPI equation^S4^. Renal histology was conducted on samples secured immediately after nephrectomy – small fragments of the kidney taken from unaffected-by-cancer pole were immersed in 10% formalin for fixation. Samples were then embedded in paraffin blocks cut into 4-µm-thick sections and stained with haematoxylin and eosin, Periodic acid-Schiff and Masson Trichrome. All samples had more than 35 glomeruli (median: 145) available for analysis and none of the samples included had structural changes consistent with diabetic or primary nephropathy. The extent of glomerulosclerosis, tubular atrophy, interstitial fibrosis and arterial/arteriolar narrowing in each sample was visually evaluated under the microscope by an experienced histopathologist and scored using a previously proposed semi-quantitative method of kidney slide evaluation. Each sample received a score on a scale 0-3 (0 – no damage).^S5^ The TRANSLATE study was ethically approved by local institutional review board and all subjects gave written informed consent for participation.

# TCGA – recruitment, sample processing and phenotypes

The Cancer Genome Atlas (TCGA)^S6^ is a publicly available repository of DNA sequence and tissue-expression from patients with more than 30 types of cancer including renal cancer. Apart from gene expression profiles of renal cancer tissues, transcriptome information is also available from “control” renal tissue. Similar to TRANSLATE Study, these specimens were taken from apparently normal kidney tissue (unaffected by cancer). Only basic demographic information including sex, ethnicity and age was available for TCGA individuals.

# RNA extraction and RNA-seq analyses in TRANSLATE Study and TCGA

In TRANSLATE Study, RNA was extracted from all kidneys using RNeasy kits (Qiagen) using previously reported protocols.^S1^ A total of 1ug of kidney RNA from TRANSLATE Study was subjected to Illumina TrueSeq RNA Sample Preparation protocol. In brief, the poly(A)-containing mRNA fraction was purified using oligo-DT magnetic beds, fragmented and subjected to first strand and second strand cDNA synthesis. The synthesised cDNA fragments underwent an end-repair, tailing with a single A-base and adapter ligation. The libraries were sequenced using either 100-base-paired-end reads (on an Illumina HiSeq 2000) or 75-base-paired-end reads (on an Illumina NextSeq or HiSeq 4000). All generated raw reads were stored in FASTQ format. The base call and read quality were evaluated using FASTQC. The input library complexity was assessed using RNA-SeQC. The pre-processing of reads for adapter trimming was conducted by Trimmomatic. The reads were then pseudo-aligned to the GRCh38 Ensembl transcriptome reference (Ensembl release 83) (chromosomal positions of all genes and variants were mapped to GRCh37 for all downstream analyses); and expression was quantified in transcripts per million (TPM) at a transcript level using Kallisto.^S7^ Transcript expression values were then summed to give gene-level quantification.

For TCGA, the details of RNA extraction, RNA-sequencing and data processing have been provided elsewhere.^S8,S9^ In brief, RNA was extracted using a modified RNA AllPrep kit (Qiagen). Libraries were generated using the Illumina TruSeq kit following manufacturers’ protocol. The sequencing was conducted on the Illumina HiSeq2000 using 50-base-paired-end reads with a mean coverage of 7.9 Gbp per sample. TCGA RNA-Seq data were downloaded from the GDC Portal^S10^ using the following criteria: “project name” – “TCGA”, “primary site” – “kidney”, “sample type” – “solid tissue normal”, “race” – “white”, “data category” – “raw sequencing data”, “data type” – “aligned reads” and “experimental strategy” – “RNA-Seq”.

## RNA-seq quality control

The same RNA-seq quality control filters were applied in both studies. Outlier samples were removed based on a statistic described in Wright *et al*.^S11^ or based on pairwise correlation between samples, where samples with median correlation <0.8 were excluded as per ‘t Hoen *et al*.^S12^ Samples without matching genotype data were also excluded. Only genes on autosomal chromosomes were selected for downstream analyses. A threshold of gene expression was set at TPM>0.1 and read counts ≥ 6 in at least 30% of samples within each study/sequencing batch. A gene was also removed if its interquartile range was zero. Only genes that passed all of the above RNA-seq quality control filters in both studies were used in downstream analyses.

In TRANSLATE Study, RNA-seq quality control was applied to 190 samples. Four outliers were excluded based on a statistic described in Wright *et al*.^S11^ and 18 more based on a statistic described in ‘t Hoen *et al*.^S12^ Three samples were excluded because of missing DNA information and 5 more were excluded based on visual examination of principal component analysis plots of normalised TPM data. This left 160 samples available for downstream analyses. Raw expression data were generated for 20244 genes. A total of 3362 genes were removed because they did not satisfy the minimum expression threshold. Additional 925 genes located on non-autosomal chromosomes were also removed. We excluded 166 genes that did not pass RNA-seq quality control filters in TCGA. The final number of kidney genes that passed all RNA-seq quality control filters was 15791.

In TCGA*,* out of 112 identified subjects, only 103 had matching genotype data. Two samples were excluded as outliers based on a statistic described in Wright *et al.**^S^*^11^ One additional sample was excluded because it failed sex check based on the expression of XIST and Y-chromosome genes. This left 100 samples available for downstream analyses. Raw expression data were available for
21623 genes. We excluded 274 genes that did not meet the minimum expression threshold. Further 1013 genes were removed because they were not located on autosomal chromosomes and one more because its interquartile range was 0. A total of 4544 genes were excluded because they did not pass the RNA-seq quality control filters in TRANSLATE Study.

## RNA-seq normalisation

Prior to any statistical analyses, gene-level TPM values were normalised in the same way in both TRANSLATE Study and TCGA. First, log_2_ of TPM values were normalised across samples using robust quantile normalisation.^S13^ Second, the normalised gene expression values were transformed using rank-based inverse normal transformation.^S14^ Third, to account for hidden variation in RNA-seq data due to technical processing (such as batch effects or sample processing in pre-sequencing stage), we used probabilistic estimation of expression residuals (PEER) method^S15^ and estimated 30 hidden factors for TRANSLATE Study and 15 for TCGA. The numbers of hidden factors were chosen based on sample sizes of each dataset as recommended in the GTEx eQTL analysis.^S16,S17^ The estimated hidden factors were used as covariates in all downstream regression analyses where appropriate.

# DNA analyses in TRANSLATE Study and TCGA

In TRANSLATE Study, DNA was extracted from homogenised kidneys using Qiagen DNeasyBlood & Tissue kit (Qiagen). The extracted DNA was genotyped using Infinium HumanCoreExome-24 BeadChip arrays. In TCGA, individuals were genotyped using Affymetrix Genome-Wide Human SNP Array 6.0; we downloaded genotype information of individuals who had matching RNA-Seq data from normal kidney tissue.

## Genotype quality control

The same set of quality control filters for genotyped markers were used in both studies. Samples were excluded if their genotyping rate was <95%, their heterozygosity rate was above/below 3 standard deviations from the mean, they had cryptic relatedness with other individuals, were of non-white European genetic ancestry or had discordant sex information (inconsistency between declared and genotyped sex). Genetic variants were excluded if their genotyping rate was <95%, they mapped to Y chromosome/mitochondrial DNA, had ambiguous chromosomal location, violated Hardy-Weinberg equilibrium (HWE) (P<0.001) or if their minor allele frequency (MAF) was <5%.

In TRANSLATE Study, the allele calls were made using Genome Studio.^S18^ Out of 603132 genotyped markers, 2789 mapped onto Y chromosome or mitochondrial DNA, 19846 had missing genotype rate >5%, 108 429 were not in HWE and 314 211 had MAF<5%. The remaining number of markers that passed all genotype quality control filters was 272343.

In TCGA, the allele calls were made using Birdseed^S19^ Out of 905306 markers genotyped on the array, 288 were on Y chromosome or mitochondrial DNA, 45369 had genotype missingness rate>5%, 702 were not in HWE and 220368 had MAF<5%. The remaining number of markers that passed all genotype quality control filters was 659711.

In both studies HWE, MAF, heterozygosity rate and variant/sample genotype missing rate were calculated using PLINK.^S20^ The sex check and analysis of genotype-derived principal components were also conducted using PLINK.^S20^ Cryptic relatedness was determined using KING.^S21^ The ancestry was investigated using SNPWeights^S22^ and EIGENSTRAT.^S23^

## Genotype principal components

Genotype principal components were calculated from genotyped data that passed all genotype quality control filters. Following eQTL analysis in the GTEx project^S16,S17,S24^, the top three principal components were used as independent variables in all analyses where appropriate. The top three principal components accounted for 16% and 17% of variation in genotypes in TRANSLATE Study and TCGA, respectively.

## Genotype imputation

Genotype imputation was conducted using minimac3^S25^ with 1000 Genomes Project's Phase 3^S26^ as the reference panel. The imputation was performed on Michigan Imputation Server.^S25^ The total number of genotyped and imputed variants was 47100201 in TRANSLATE Study and 47101134 in TCGA. Post-imputation, we excluded duplicate variants, non-SNPs, variants with low imputation coefficient (R^2^<0.4), low frequency variants (MAF<5%) or those violating HWE (P<10^-6^).

A total of 160 samples from TRANSALTE Study and 100 samples from TCGA were included in the meta-eQTL analysis with a total of common 5689943 genetic variants and common 15791 genes that passed all quality control filters. The details of exclusion of imputed markers at each stage of quality control are shown in TabS23.

Since there were only 94 samples with DNA methylation data in TRANSLATE Study, the post-imputation quality control for mQTL analysis was re-checked prior to mQTL analysis. A total of
5689943 variants passed all post-imputation quality control filters as shown in TabS24. MAF and HWE were recalculated for appropriate subsets of samples using HardyWeinberg R package.^S27^

## DNA methylation

To determine the pattern of 5-methylcytosine residues in kidney DNA we used 96 TRANSLATE Study renal DNA samples (750 ng). DNA underwent bisulphite conversion with the use of the Zymo EZ DNA Methylation Kit. The converted DNA samples (4μL, at 50ng/μL concentration) were then hybridised with Illumina HumanMethylation450 BeadChip array. The degree of methylation at each site was measured as an M-value – log_2_ of a ratio between methylated probe and unmethylated probe intensities.

Out of 96 TRANSLATE Study individuals whose kidney DNA was hybridised to HumanMethylation 450 BeadChip array, 2 were excluded because of sex information inconsistency between DNA methylation data and the reported sex data. One individual was excluded because of missing clinical information. All remaining 93 samples had call rate (calculated based on detection P-value threshold of 1x10^-16^) of at least 98%.

Out of the 485512 probes, 15311 probes were excluded due to a call rate below 95% (based on detection P-value threshold of 1x10^-16^). 11648 probes on X/Y chromosomes, 29233 cross-reactive probes and 17302 probes containing common SNPs (MAF≥1%) were also excluded. This left
418581 probes available for downstream analyses.

DNA methylation data that passed the above quality control filters was processed using “dasen” method from wateRmelon R package.^S28^ Each consonant letter in “dasen” stands for a specific type of data normalisation: “d” – background adjustment by adding the offset between Type I and Type II probe intensities to Type I intensities, “s” – between-sample quantile normalisation applied to Type I and Type II probes separately and “n” – indicating no dye bias adjustment (the two vowels, “a” and “e”, were added by the authors for ease of pronunciation). In addition to wateRmelon R package, the following R packages were used for pre-processing DNA methylation data: minfi^S29^ and missMethyl.^S30^

Kidney DNA methylation in TCGA population was measured using Infinium Human Methylation 450K BeadChip. The extent of methylation at CpG sites was quantified using β-values.^S31^ For the purpose of the age-TSPYL5 promoter methylation replication analysis we included 126 apparently normal “control” kidney samples with matching chronological age, sex and DNA methylation data.

# *Nephroseq* – kidney gene expression profiles

We used Nephroseq^S32^ – a platform for integrative data mining of comprehensive renal disease gene expression datasets – as a replication resource. A total of 303 kidney samples from renal cortex/glomerulus and 299 kidney samples from medulla/tubulointerstitial compartment from three eligible studies by Rodwell *et al*.^S33^, Ju *et al*.^S34^ and Sampson *et al*.^S35^ were available for analysis of association between age and gene expression. The kidney gene expression profiles were originally generated in those studies using microarrays; the data were then deposited in and re-processed by *Nephroseq* facilitating analysis of association between individual genes and demographic traits (including age).

# GTEx project – recruitment, sample processing and phenotypes

We used gene expression profiles from 42 tissues obtained from 532 individuals recruited in the Genotype-Tissue Expression project (GTEx).^S16,S24^ In brief, tissues were preserved using the PAXgene Tissue preservation system (Qiagen) and RNA and DNA tissue-specific extraction protocols were followed^S16,S24^. RNA were sequenced using Illumina TruSeq 75-base-paired-end sequencing RNA protocol, averaging ~50 million aligned reads per sample. The detailed GTEx sample inclusion criteria were outlined in previous studies^S36^. We excluded GTEx tissues with a sample count <50 from our analysis. Similar to previous GTEx analyses, only genes with expression of TPM>0.1 and read counts ≥6 in 20% of samples were selected for the analysis. The log_2_-transformed TPM values were first quantile-normalised and then transformed using rank-based inverse normal transformation. The number of PEER hidden factors was determined for each tissue individually based on its sample size, as recommended by GTEx.

# TSPYL5 – human kidney immunohistochemistry

Tissues were fixed in 4% paraformaldehyde, embedded in paraffin and sectioned at 5 µm. Sections were de-waxed, rehydrated and boiled in an 800W microwave in 10 mM sodium citrate buffer (pH 6.0). After cooling to room temperature, endogenous peroxidase activity was blocked using 0.3% H_2_O_2_ in PBS for 10 minutes. Sections were permeabilized using 0.2% Triton X-100 (Sigma-Aldrich) for 10 minutes and blocked using 1% bovine serum albumin (BSA) with 10% rabbit serum. Sections were then incubated overnight at 4°C with the primary antibody in 1% BSA. Two rabbit polyclonal anti-TSPYL5 antibodies Abcam (1 in 200 dilution, ab203657, raised against human TSPYL5 aa 263-302) and Sigma (1:5000 dilution, HPA031347 raised against human TSPYL5 sequence SQEKEVLSYLNSLEVEELGLARLGYKIKFYFDRNPYFQNKVLIKEYGCGPSGQVVSRSTPIQWLPGHDLQSLSQGNPENNRSFFGWFSNHSSIESDKIVEIINEELWPNPLQFYLLSEGARV) were tested and both showed similar staining pattern: results using the Sigma antibody are depicted in the Figure. Negative controls omitted the primary antibodies. Primary antibodies were detected, and imaging undertaken, as described before^S37^. Biotin-conjugated species-specific secondary antibodies with 1% BSA were incubated at room temperature for two hours. Following PBS washes, slides were incubated in avidin-biotin enzyme complex (Vector Laboratories VECTASTAIN Elite ABC Reagent, PK-6100) for one hour at room temperature. Peroxidase activity was detected with the 3, 3'-diaminobenzidine (DAB) peroxidase substrate solution (Vector Laboratories, SK4100) followed by hematoxylin counterstaining. Sections were dehydrated and mounted with DPX mounting medium. Images were collected on an Olympus BX63 upright microscope. For intensity analysis, images were acquired on a 3D-Histech Pannoramic-250 microscope slide-scanner (Zeiss) and the selected images were captured using the Panoramic Viewer software. Images were then processed and analysed using ImageJ (http://imagej.net/Fiji/Downloads). Images were submitted to the “colour deconvolution” plug-in using the built-in vector HDAB, where the stainings of hematoxylin and DAB were separated into 3 different panels with hematoxylin only, with DAB only and the background image. From DAB image the areas to be analysed were highlighted by adjusting the threshold. The software calculated the mean value of DAB, ranging from 0 (black) to 255 (total white). The final DAB intensity was calculated using the formula f = 255 − i, where f is the final DAB intensity, i is mean DAB intensity; i ranges from 0 to 255 (zero being highest expression and darkest brown and 255 being the total white)^S38^. The statistical significance of the difference in kidney TSPYL5 immunostaining between individuals aged 60 years or older and those younger than 60 years was examined by a Mann-Whitney U.

# Statistical and bioinformatic analyses

## Discovery analysis

Associations between age and kidney gene expression in the discovery analysis were examined using multiple linear regression by regressing normalised gene expression on age, sex, the top three genotype principal components and PEER hidden factors. The regression models were fitted separately to TRANSLATE Study and TCGA data. The results from each study were then combined through inverse variance meta-analysis and corrected for multiple testing using Storey’s method^S39^ (qvalue R package^S40^) with a threshold of 5%.

## Replication analysis

The association between renal expression of each gene implicated in the discovery analysis and age was examined in *Nephroseq* using Pearson's correlation coefficient. The coefficients from three eligible studies were combined using Olkin-Pratt fixed-effect meta-analysis approach. This was carried out separately for kidney cortex/glomerular and kidney medulla/tubulointerstitial samples. Correction for multiple testing was based on Benjamini-Hochberg method within each meta-analysis. A gene was considered replicated if it survived an FDR threshold of 5% in at least one of the two meta-analyses.

In sensitivity analyses, we conducted a separate replication analyses for samples with apparently normal renal tissue (72 from glomerular compartment/cortex and 62 from tubulointerstitial compartment/medulla; Rodwell *et al*.^S33^) and measured the extent to which the replicated genes overlapped with the genes replicating in kidney specimen collected from patients with kidney disease (231 from glomerular compartment/cortex and 237 tubulointerstitial compartment/medulla; Ju *et al*.^S34^ and Sampson *et al*.^S35^). Of 31 age-associated genes available for replication in the Rodwell’s dataset deposited in *Neproseq* 10 were associated with age (in the same direction as the discovery cohort) after a correction for multiple testing (at FDR of 5%). 60% of these overlapped with age-associated genes identified at the replication stage restricted to samples from patients with kidney disease.

## GTEx non-renal tissue analysis

The analysis of association of gene expression with age in 42 non-renal GTEx tissues^S16,S24^ was carried out in 532 individuals of white European ancestry using the same methodology as in individual TRANSLATE and TCGA studies. After identifying age-associated genes within each non-renal GTEx tissue, we examined their overlap with our age-associated kidney genes. The sensitivity analyses were conducted using different numbers of PEER-derived factors.

## Renal function and structure analysis

The replicated age-related genes were examined for association with eGFR and four histological measures of kidney damage (glomerular sclerosis, tubular atrophy, interstitial fibrosis and arterial/arteriolar narrowing) in TRANSLATE Study. The analysis was conducted using multiple linear regression by regressing normalised gene expression on respective renal phenotype, sex, the top three genotype principal components and 30 PEER-estimated hidden factors. Statistical significance was decided based on FDR< 5% determined using Benjamini-Hochberg method. The sensitivity analysis were conducted using age as an additional covariate.

## Cross-species validation of age-related human kidney genes in mice

We exploited kidney gene expression profiles measured at several time points (13, 26, 52, 78, 104 and 130 weeks) in C57BL/6J mice as an in silico cross-species validation analysis for 19 genes showing renal expression change with age in humans. In brief, the murine data were generated and published before as an *in vivo* study of gene expression changes in five organs across the entire murine life span.^S41^ We have first identified a mouse homolog for each of 19 age-related human genes and examined its presence on the gene expression microarray/availability for in silico cross-species validation. We then explored the association between the available murine homologs and chronological age. For the homologs associated with age we further examined their association with two measures of age-related kidney involution (glomerular membrane thickening and tubular degeneration) using the data generated by Jonker MJ et al.^S41^ We considered a nominal level of statistical significance for association with age or kidney histology as consistent with cross-species validation.

## Association between renal expression of TSPYL5 and age in rats

A total number of 21 normal heart rats (NHR) were sacrificed^S42^ at 3 different time points; 2 days (3 males and 3 females), 13 weeks (5 males and 5 females) and 33 weeks (3 males and 2 females). Kidneys were immediately harvested, preserved in liquid nitrogen and transferred to a 80°C freezer. Kidney RNA was extracted using TRIzol^TM^ reagent (Invitrogen) following manufacturer’s instructions. cDNA synthesis was conducted using the High Capacity cDNA Reverse Transcription Kit (ThermoFisher Scientific). TSPYL5 and glyceraldehyde-3-phosphate dehydrogenase (GAPDH) primers were designed using NCBI Primer Blast. Amplification reactions were performed in duplicates using SensiFASTTM Lo-Rox® SYBR (Bioline) in the ViiA 7 qPCR instrument (Life Technologies). The statistical significance of differences in absolute abundance of TSPYL5 mRNA between different ages was conducted by ANOVA. The studies were approved by the Animal Ethics Committees of the University of Melbourne and Deakin University and ratified at Federation University, Australia.

## cis-eQTL meta-analysis

The association between gene expression and genotype was conducted using multiple linear regression with normalised gene expression as the dependent variable and genotype dosage, sex, top three genotype-derived principal components and PEER-estimated hidden factors (30 for TRANSLATE Study and 15 for TCGA) as independent variables. These analyses were conducted in both TRANSLATE Study and TCGA separately. The estimated coefficients from both studies were meta-combined using inverse variance method.^S43^ Each of the selected age-related genes was examined for association with all variants within 1 Mb from the closest boundary of the gene. Correction for multiple testing for analysis of each robust age-related genes with all in*-cis* SNPs was conducted using permutations, where the distribution of the smallest meta-combined P-value was determined using 2000 permutations. At each permutation, the genotype sample labels were permuted but kept coupled with the sample labels of the top three genotype principal components. For each gene, the associations between its expression and SNPs were re-estimated and the smallest meta-combined P-value recorded. Finally, for each robust age-associated gene the SNP with the smallest meta-combined P-value was identified and adjusted using the corresponding empirical distribution of the smallest meta-combined P-values for that gene. False discovery rate was determined using Storey’s method^S39^ (qvalue R package^S40^). The permutation corrected P-values were used for calculating FDR.

A threshold for nominal meta-combined P-values for SNPs that did not have the smallest meta-combined P-values was calculated as follows. First, a global permutation P-value, p_t_, was chosen to be the permutation P-value for the gene whose permutation P-value was closest to FDR 5%. Then for each gene, a threshold for meta-combined nominal P-values was chosen to be the probability of observing a meta-combined P-value less than or equal to p_t_ using the gene's empirical distribution of the smallest meta-combined P-values. This method is similar to the one used in the GTEx eQTL analysis.^S17^

In total, there were 4217 genes that passed the multiple test correction using an FDR threshold of 5% and 470198 statistically significant gene-SNP pairs based on the nominal P-value cut-offs. Out of the 19 age-associated-replicated genes, four had FDR below 5% and 78522 SNPs had a statistically significant association with the expression of at least one of these four genes.

## Association between age-related eSNPs and estimated glomerular filtration rate – CKDGen Consortium

Summary statistics of CKDGen GWAS^S44^ were downloaded from http://ckdgen.imbi.uni-freiburg.de and we examined associations between the identified eSNPs for the ageing genes (PPP1R3C, TSPYL5, LYG1 and LTF) and eGFR. Genotype and phenotype information was available for 110527 individuals of white European ancestry. Given the tight linkage disequilibrium between eSNPs for each of the genes, we considered all nominal associations between eSNPs and eGFR as statistically significant.

## DNA methylation and gene expression analysis

The association between renal expression of robust age-associated genes and renal DNA methylation in TRANSLATE Study was evaluated using multiple linear regression. The models were constructed using normalised gene expression as the dependent variable and M-values of CpG site methylation levels, sex, the top three genotype-derived principal components, smoking status (ever-smoker or never-smoker), and 30 PEER-estimated hidden factors as the independent variables.

The analysis was carried out on 78 samples that had both gene expression and DNA methylation data. Only CpG sites within 1000 base-pair distance from the closest boundary of the gene were included in the analysis. The correction for multiple testing of each gene against its in*-cis* CpG sites was calculated using 2000 permutations. At each permutation, sample labels of M-values were randomly re-arranged, the association between gene expression and M-values for each in*-cis* CpG site re-calculated and the smallest P-value recorded. The resulting set of 2000 smallest P-values with the observed smallest P-value were used to derive an empirical distribution of the smallest P-value for each gene. Then, for each gene, the permutation P-value was derived from its corresponding empirical distribution of the smallest P-value as the probability of obtaining a P-value as small as the smallest P-value observed for the gene. To further correct for the number of genes examined, Benjamini-Hochberg method was applied on permutation-derived P-values.

## DNA methylation and age analysis

Analysis of relationship between methylation of age-associated renal genes and age in TRANSLATE Study was based on multiple linear regression models constructed using M-values of CpG site methylation as the dependent variable and age, sex, smoking status (ever-smoker or never-smoker) and the top three genotype principal components as independent variables. Correction for multiple testing was based on Bonferroni method. Only three CpG sites related to one gene, which survived the correction for multiple testing in the previous analysis were considered. The analysis of association between age and methylation of TSPYL5 promoter at cg22328208 site in TCGA was conducted using multiple regression model whereby logarithmically transformed methylation β-values were the dependent variable while age and sex were independent variables.

## mQTL analysis

Association of DNA methylation with genotype (mQTL analysis) was investigated using multiple linear regression, where M-values of CpG site methylation were the dependent variable while genotype dosage, sex, smoking status (ever-smoker or never-smoker) and the top three genotype principal components were the independent variables. Only a single CpG site most strongly associated with age-related gene expression was included in this analysis. This CpG site was examined against all genotype variants within 33172 base-pairs on either side. Correction for multiple testing was carried out using the Benjamini-Hochberg method with a cut-off of 5%.

## Causality analysis

We applied causal inference test^S45^ to investigate whether the effects of the best eSNP and mSNP mediate the changes on renal gene expression through changes in CpG methylation (epigenetic mediation model) or whether their effects on methylation are mediated through the changes in gene expression (transcriptional mediation model). We also used mediation analysis to explore whether the effect of age is driving the gene expression through its effect on DNA methylation or whether age acts on DNA methylation through its effect on renal gene expression. Specifically, four associations were examined: i) the SNP is associated with the potential mediator; ii) the SNP is associated with the outcome; iii) the SNP is associated with the potential mediator conditional on the outcome and iv) the SNP is independent with the outcome conditional on the potential mediator. A P-value (two-tailed) was assigned to each set of associations using the intersection-union test^S45^ Bonferroni correction was applied to account for the number of tested association sets (n=3) and the corrected level of significance was calculated at 0.017. We also used Mendelian randomization (inverse variance weighting, weighted median) to evaluate whether the magnitude of DNA methylation is causal to the level of renal gene expression.^S46^ Then MR-Egger regression was used to test direction pleiotropy as a sensitivity analysis. Six independent SNPs were selected as instruments for the analysis. The level of statistical significance was set at 0.05.

## Bioinformatic analysis

Functional characterisation *in silico* of genes associated with ageing was conducted using data from Ensembl GRCh37 (release 91). Genes were annotated with molecular, biological and cellular labels using data from the Gene Ontology repository.^S47^ In addition to using data from GTEx, we searched the Human Protein Atlas^S48^ to investigate the organ-specificity of gene expression for the age-associated renal genes. Genes previously associated with human ageing were identified in the GenAge Database.^S49^ Previously documented age-associated transcriptomic data from other organ studies was obtained using the Digital Ageing Atlas.^S50^ The Mouse Genome Informatics database^S51^ was searched for phenotypic traits associated with knockout or knockdown of the gene of interest. Furthermore, Mendelian traits associated with each gene were identified in a search of the Online Mendelian Inheritance in Man repository.^S52^ Finally, we used the Drug–Gene Interaction Database^S53^ to identify potential drug-gene interactions, which represent mechanisms by which to clinically interact with gene activity.

ChIP-seq signal data for 4 different histone modifications in adult kidney tissue from Roadmap Epigenomics (H3K4me1, H3K4me3, H3K36me3, H3K9me3) were binarised and combined into a single chromatin state segmentation using ChromHMM^S54^ following the standard Roadmap Epigenomics protocol^S55^ for the 15-state segmentation. The 15-state model file from Roadmap was used for the final segmenation by ChromHMM. Raw ChIP-seq read data was downloaded from Roadmap Epigenomics (GEO Series GSE19465) and smoothed across the relevant locus. Human chromatin interaction data was downloaded from 4DGenome.^S56^ All interacting chromatin regions proximal to TSPYL5 were identified and assessed by filtering the database through a contact frequency of greater than 5 (more than 5 ChIP-seq reads supporting the interaction) and an interaction confidence score of <0.05 (adjusted binomial interaction test p-value <0.05).

# References

S1. Marques F.Z, Romaine S.P, Denniff M, et al. Signatures of mir-181a on the renal transcriptome and blood pressure. *Mol. Med*. 2015;21:739–748.

S2. Tomaszewski M, Eales J, Denniff M, et al. Renal mechanisms of association between fibroblast growth factor 1 and blood pressure. *JASN*. 2015;26:3151–3160.

S3. Tomaszewski M, Charchar F.J, Nelson C.P, et al. Pathway analysis shows association between FGFBP1 and hypertension. *JASN*. 2011;22:947–955.

S4. Levey A.S, Stevens L.A, Schmid C.H, et al. A new equation to estimate glomerular filtration rate. *Ann. Intern. Med.* 2009;150:604–612.

S5. Remuzzi G, Grinyo J, Ruggenenti P, et al. Early experience with dual kidney transplantation in adults using expanded donor criteria. *JASN*. 1999;10:2591–2598.

S6. Chang K, Creighton C.J, Davis C, et al. The Cancer Genome Atlas Pan-Cancer analysis project. *Nat. Genet*. 2013;45:1113–1120.

S7. Bray N.L, Pimentel H, Melsted P, et al. Near-optimal probabilistic RNA-seq quantification. *Nat. Biotechnol*. 2016;34:525–527.

S8. Peng L, Bian X.W, Li D.K, et al. Large-scale RNA-Seq transcriptome analysis of 4043 cancers and 548 normal tissue controls across 12 TCGA cancer types. *Sci. Rep*. 2015;5:13413.

S9. Cancer Genome Atlas Research Network, Linehan W.M, Spellman P.T, et al. Comprehensive molecular characterization of papillary renal-cell carcinoma. *N. Engl. J. Med*. 2016;374:135–145.

S10. Genomic Data Commons Data Portal:https://portal.gdc.cancer.gov/ accessed on 01/10/2017.

S11. Wright F.A, Sullivan P.F, Brooks A.I, et al. Heritability and genomics of gene expression in peripheral blood. *Nat. Genet*. 2014;46:430–437.

S12. ’t Hoen P.A.C, Friedländer M.R, Almlöf J, et al. Reproducibility of high-throughput mRNA and small RNA sequencing across laboratories. *Nat. Biotechnol*. 2013;31:1015–1022.

S13. Bengtsson, H. aroma - An R Object-oriented Microarray Analysis environment (Sweden: Mathematical Statistics, Centre for Mathematical Sciences, Lund University).

S14. Karssen LC, van Duijn CM, Aulchenko YS. The GenABEL Project for statistical genomics. *F1000Res*. 2016;5:914

S15. Stegle O, Parts L, Piipari M, et al. Using probabilistic estimation of expression residuals (PEER) to obtain increased power and interpretability of gene expression analyses. *Nat. Protoc*. 2012;7:500–507.

S16. GTEx Consortium. The Genotype-Tissue Expression (GTEx) pilot analysis: Multi-tissue gene regulation in humans. Science. 2015;348:648–660.

S17. GTEx Portal:https://www.gtexportal.org accessed on 01/11/2017.

S18. Genome Studio: <https://emea.illumina.com/techniques/microarrays/array-data-analysis-experimental-design/genomestudio.html>accessed on 01/01/2016.

S19. Birdseed: http://archive.broadinstitute.org/mpg/birdsuite/birdseed.html accessed on 01/11/2017.

S20. Purcell S, Neale B, Todd-Brown K, et al. PLINK: a tool set for whole-genome association and population-based linkage analyses. *Am. J. Hum. Genet*. 2007;81:559–575.

S21. Manichaikul A, Mychaleckyj J.C, Rich S.S, et al. Robust relationship inference in genome-wide association studies. *Bioinforma. Oxf. Engl*. 2010;26:2867–2873.

S22. Chen C.Y, Pollack S, Hunter D.J, et al. Improved ancestry inference using weights from external reference panels. *Bioinformatics*. 2013;29:1399–1406.

S23. Price, A.L, Patterson, N.J, Plenge, R.M, et al. Principal components analysis corrects for stratification in genome-wide association studies. *Nat. Genet.* 2006; 38: 904.

S24. GTEx Consortium. The Genotype-Tissue Expression (GTEx) project. *Nat. Genet.* 2013;45:580–585.

S25. Das S, Forer L, Schönherr S, et al. Next-generation genotype imputation service and methods. *Nat. Genet*. 2016;48:1284–1287.

S26. The 1000 Genomes Project Consortium. A global reference for human genetic variation. *Nature*. 2015;526:68–74.

S27. Graffelman J, and Camarena J.M. Graphical Tests for Hardy-Weinberg Equilibrium Based on the Ternary Plot. *Hum. Hered*. 2008;65:77–84.

S28. Pidsley R, Y Wong C.C, Volta M, et al. A data-driven approach to preprocessing Illumina 450K methylation array data. *BMC Genomics*. 2013;14:293.

S29. Aryee M.J, Jaffe A.E, Corrada-Bravo H, et al. Minfi: a flexible and comprehensive Bioconductor package for the analysis of Infinium DNA methylation microarrays. *Bioinformatics*. 2014;30:1363–1369.

S30. Phipson B, Maksimovic J, and Oshlack A. missMethyl: an R package for analyzing data from Illumina’s HumanMethylation450 platform. *Bioinforma. Oxf. Engl*. 2016;32:286–288.

S31. Tomczak, K., Czerwińska, P. & Wiznerowicz, M. The Cancer Genome Atlas (TCGA): an immeasurable source of knowledge. *Contemp. Oncol.* 2015;19:A68–A77.

S32. Nephroseq: <https://www.nephroseq.org/resource>accessed on 01/11/2017.

S33. Rodwell G.E.J, Sonu R, Zahn J.M, et al. A transcriptional profile of aging in the human kidney. *PLoS Biol*. 2004;2:e427.

S34. Ju W, Nair V, Smith S. et al. Tissue transcriptome-driven identification of epidermal growth factor as a chronic kidney disease biomarker. *Sci. Transl. Med.* 2015;7:316ra193.

S35. Sampson M.G, Robertson C.C, Martini S, et al. Integrative genomics identifies novel associations with apol1 risk genotypes in black neptune subjects. *JASN.* 2016;27:814–823.

S36. Carithers L.J, Ardlie K, Barcus M, et al. A novel approach to high-quality postmortem tissue procurement: the gtex project. *Biopreservation Biobanking*. 2015;13:311–319.

S37. Bantounas, I. *et al.* Generation of functioning nephrons by implanting human pluripotent stem cell-derived kidney progenitors. *Stem Cell Rep.* 2018;10:766–779.

S38. Nguyen D. Quantifying chromogen intensity in immunohistochemistry via reciprocal intensity. *Protocol Exchange.* 2013. doi:10.1038/protex.2013.097

S39. Storey J.D, Taylor J.E, and Siegmund, D. Strong control, conservative point estimation and simultaneous conservative consistency of false discovery rates: a unified approach. *J. R. Stat. Soc. Ser. B Stat. Methodol*. 2004;66:187–205.

S40. Storey JD. A direct approach to false discovery rates. *Journal of the Royal Statistical Society*. 2012; Series B, 64:479-498.

S41. Jonker, M. J. *et al.* Life spanning murine gene expression profiles in relation to chronological and pathological aging in multiple organs. *Aging Cell.* 2013;12:901–909.

S42. Marques, F. Z. *et al.* Telomere dynamics during aging in polygenic left ventricular hypertrophy. *Physiol. Genomics.* 2016;48:42–49.

S43. Willer C.J, Li Y, and Abecasis G.R. METAL: fast and efficient meta-analysis of genomewide association scans. *Bioinformatics*. 2010;26:2190–2191.

S44. Gorski, M. *et al.* 1000 Genomes-based meta-analysis identifies 10 novel loci for kidney function. *Sci. Rep.2017*;7.

S45. Millstein J, Zhang B, Zhu J, et al. Disentangling molecular relationships with a causal inference test. *BMC Genet*. 2009;10:23.

S46. Bowden J, Davey Smith G, Haycock P.C, et al. Consistent estimation in mendelian randomization with some invalid instruments using a weighted median estimator. *Genet. Epidemiol*. 2016;40:304–314.

S47. Ashburner M, Ball C.A, Blake J.A, et al. Gene ontology: tool for the unification of biology. The Gene Ontology Consortium. *Nat. Genet*. 2000;25:25–29.

S48. Pontén F, Jirström K, and Uhlen M. The Human Protein Atlas--a tool for pathology. *J. Pathol*. 2008;216:387–393.

S49. de Magalhães J.P, and Toussaint O. GenAge: a genomic and proteomic network map of human ageing. *FEBS Lett*. 2004;571:243–247.

S50. Craig T, Smelick C, Tacutu R, et al. The Digital Ageing Atlas: integrating the diversity of age-related changes into a unified resource. *Nucleic Acids Res*. 2015;43:D873-878.

S51. Blake J.A, Richardson J.E, Bult C.J, et al. MGD: the Mouse Genome Database. *Nucleic Acids Res.* 2003;31:193–195.

S52. Hamosh A, Scott A.F, Amberger J, et al. Online Mendelian Inheritance in Man (OMIM), a knowledgebase of human genes and genetic disorders. *Nucleic Acids Res.* 2002;30:52–55.

S53. Griffith M, Griffith O.L, Coffman A.C, et al. DGIdb: mining the druggable genome. *Nat. Methods*. 2013 10:1209–1210.

S54. Ernst J, and Kellis M. Chromatin-state discovery and genome annotation with ChromHMM. *Nat. Protoc*. 2017;12:2478–2492.

S55. Kundaje A, Meuleman W, Ernst J, et al. Integrative analysis of 111 reference human epigenomes. *Nature*. 2015;518:317–330.

S56. Teng, L., He, B., Wang, J. & Tan, K. 4DGenome: a comprehensive database of chromatin interactions. *Bioinformatics*.2015;31:2560–2564.
