# Supplementary material for: Uncovering genetic mechanisms of kidney aging through transcriptomics, genomics, and epigenomics
Source: Kidney Int. 2019 Mar;95(3):624–35. doi: 10.1016/j.kint.2018.10.029 (PMC6390171; doi:10.1016/j.kint.2018.10.029)
Supplement: Figure S1 — Analysis of association between age and kidney gene expression, a meta-analysis of the TRANScriptome of renaL humAn TissuE (TRANSLATE) Study and The Cancer Genome Atlas (TCGA). β, a coefficient of regression from the meta-analysis, is plotted on the X axis, statistical significance [-log10 (P value)] is plotted on the Y axis (inversely log-transformed for ease of interpretation), the dotted line shows a threshold for correction for multiple testing [false discovery rate (FDR), 5%], genes upregulated with age at FDR <0.05 are shown in green, genes down-regulated with age at FDR < 0.05 are shown in red. [file mmc2.docx]

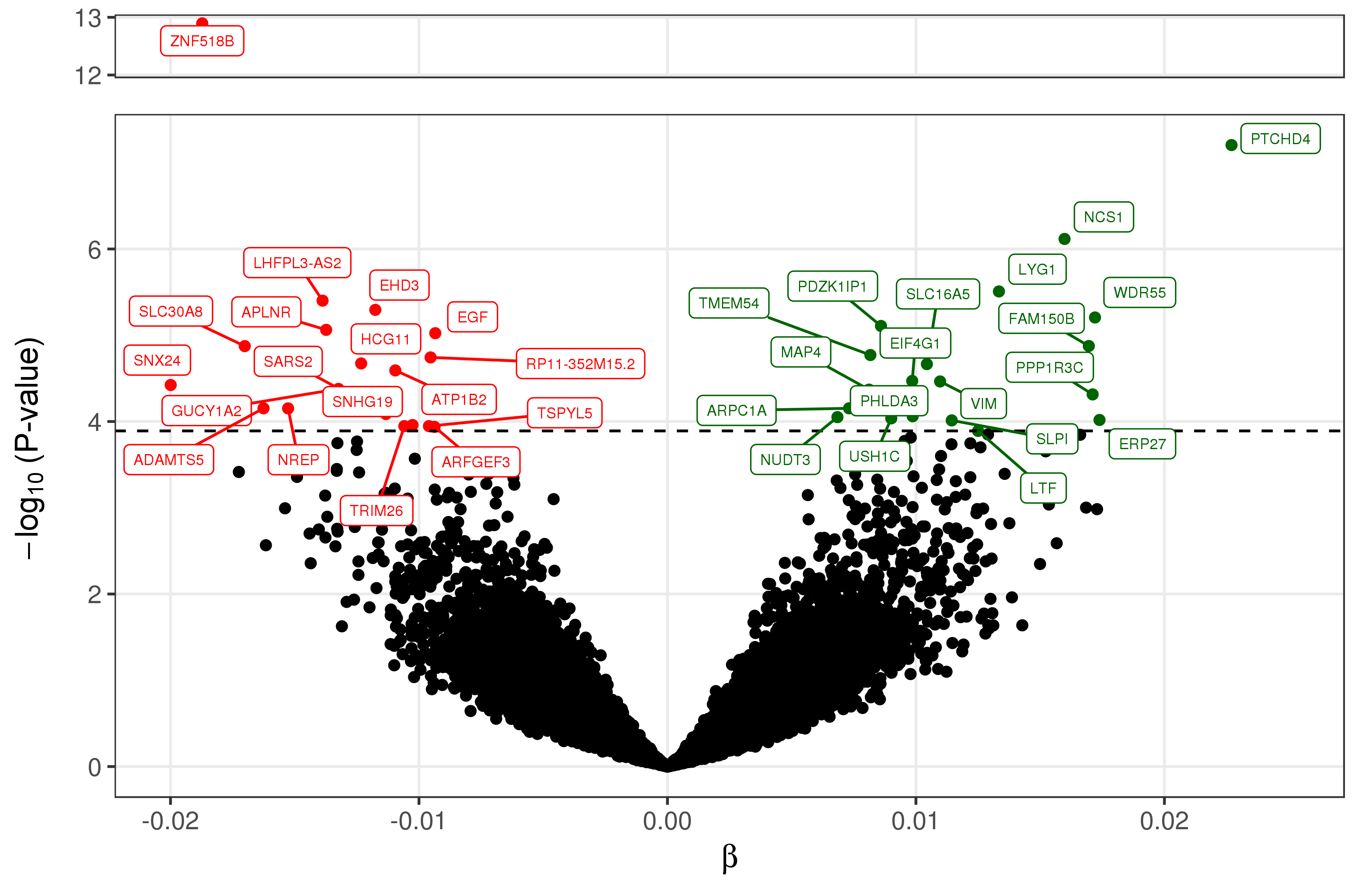


# Figure S1. Analysis of association between age and kidney gene expression – meta-analysis of TRANSLATE Study and TCGA.

β – a coefficient of regression from the meta-analysis is plotted on the X axis, statistical significance [-log10(P-value)] is plotted on the Y axis (inversely log- transformed for ease of interpretation), the dotted line depicts a threshold for correction for multiple testing [false discovery rate (FDR) of 5%], genes up-regulated with age at FDR<0.05 are depicted in green, genes down-regulated with age at FDR<0.05 are depicted in red.
