# Supplementary material for: Uncovering genetic mechanisms of kidney aging through transcriptomics, genomics, and epigenomics
Source: Kidney Int. 2019 Mar;95(3):624–35. doi: 10.1016/j.kint.2018.10.029 (PMC6390171; doi:10.1016/j.kint.2018.10.029)
Supplement: Figure S2 — Analysis of association between replicated age-associated genes and 5 measures of renal health in the TRANScriptome of renaL humAn TissuE (TRANSLATE) Study. Different colors show the magnitude of statistical significance for association between the expression of each individual gene and a given phenotype. Row 1: Genes whose expression increased with age in the TRANSLATE Study/The Cancer Genome Atlas (TCGA) analysis are shown in dark green. Genes whose expression decreased with age in the TRANSLATE Study/TCGA analysis are shown in red. Rows 2 to 6: Genes whose expression is associated positively with the given renal phenotype after correction for multiple testing [false discovery rate (FDR), < 5%] are shown in dark green. Genes whose expression is associated positively with the given renal phenotype at the nominal level (P < 5%) are shown in light green. Genes whose expression is associated inversely with the given renal phenotype after correction for multiple testing (FDR, <5%) are shown in red. Genes whose expression is associated inversely with the given renal phenotype at the nominal level (P < 5%) are shown in orange. Genes whose expression was not associated significantly with the given renal phenotype are shown in gray. The estimated glomerular filtration rate (eGFR) was calculated based on the EPI-CKD formula. [file mmc3.docx]

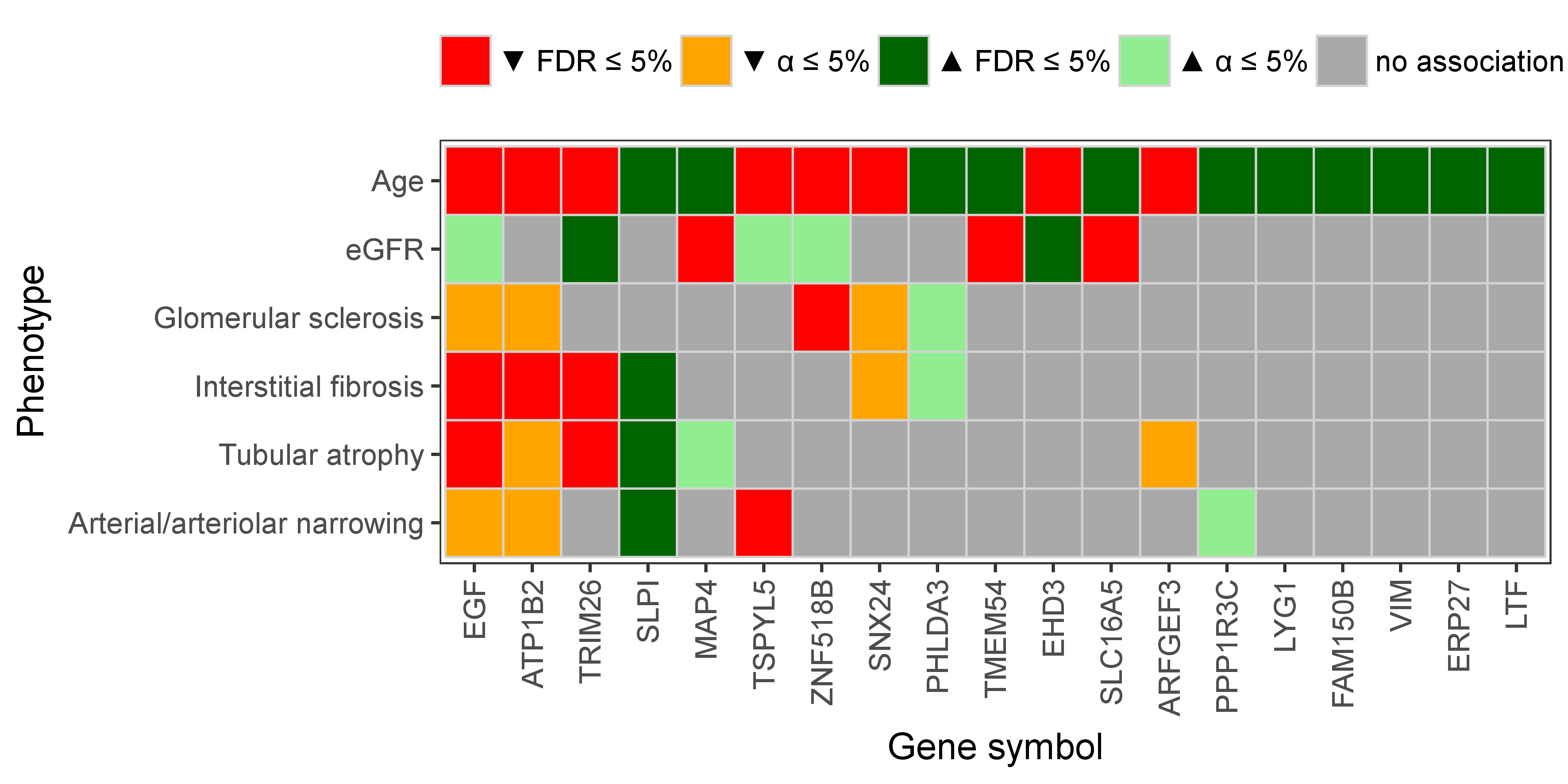


# Figure S2. Analysis of association between replicated age-associated genes and 5 measures of renal health in TRANSLATE Study.

Different colours show the magnitude of statistical significance for association between the expression of each individual gene and a given phenotype. Row 1: Genes whose expression increased with age in the TRANSLATE Study/TCGA analysis are coloured in dark green. Genes whose expression decreased with age in the TRANSLATE Study/TCGA analysis are coloured in red. Rows 2-6: Genes whose expression is positively associated with the given renal phenotype after correction for multiple testing (FDR<5%) are coloured in dark green. Genes whose expression is positively associated with the given renal phenotype at the nominal level (P-value <5%) are coloured in light green. Genes whose expression is inversely associated with the given renal phenotype after correction for multiple testing (FDR <5%) are coloured in red. Genes whose expression is inversely associated with the given renal phenotype at the nominal level (P-value <5%) are coloured in orange. Gene whose expression is not significantly associated with the given renal phenotype are coloured in grey, eGFR – estimated glomerular filtration rate calculated based on EPI-CKD formula.
