# Supplementary material for: Uncovering genetic mechanisms of kidney aging through transcriptomics, genomics, and epigenomics
Source: Kidney Int. 2019 Mar;95(3):624–35. doi: 10.1016/j.kint.2018.10.029 (PMC6390171; doi:10.1016/j.kint.2018.10.029)
Supplement: Figure S3 — Association of renal expression of TSPYL5 with age in rats. P value: level of statistical significance from analysis of variance (ANOVA). [file mmc4.docx]

**
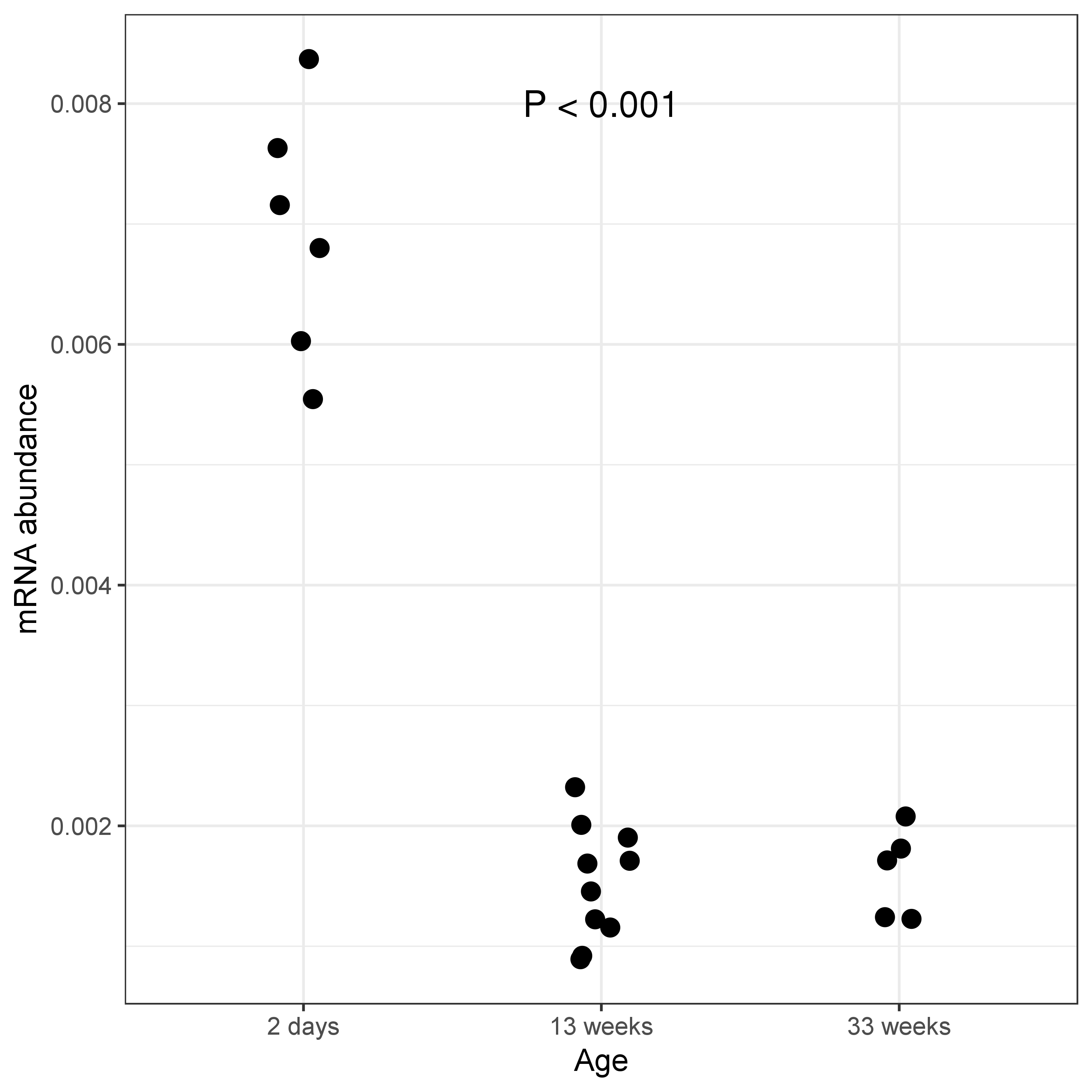
**

# Figure S3. Association of renal expression of TSPYL5 with age in rats.

P-value: level of statistical significance from ANOVA
