# Supplementary material for: Uncovering genetic mechanisms of kidney aging through transcriptomics, genomics, and epigenomics
Source: Kidney Int. 2019 Mar;95(3):624–35. doi: 10.1016/j.kint.2018.10.029 (PMC6390171; doi:10.1016/j.kint.2018.10.029)
Supplement: Figure S4 — Analysis of the difference in immunohistochemistry-derived signal intensity for kidney TSPYL5 between younger (age, ≤60 yr) and older (age, >60 yr) individuals from the TRANScriptome of renaL humAn TissuE (TRANSLATE) Study. N, number of individuals; P value, level of statistical significance using the Mann–Whitney U test. [file mmc5.docx]

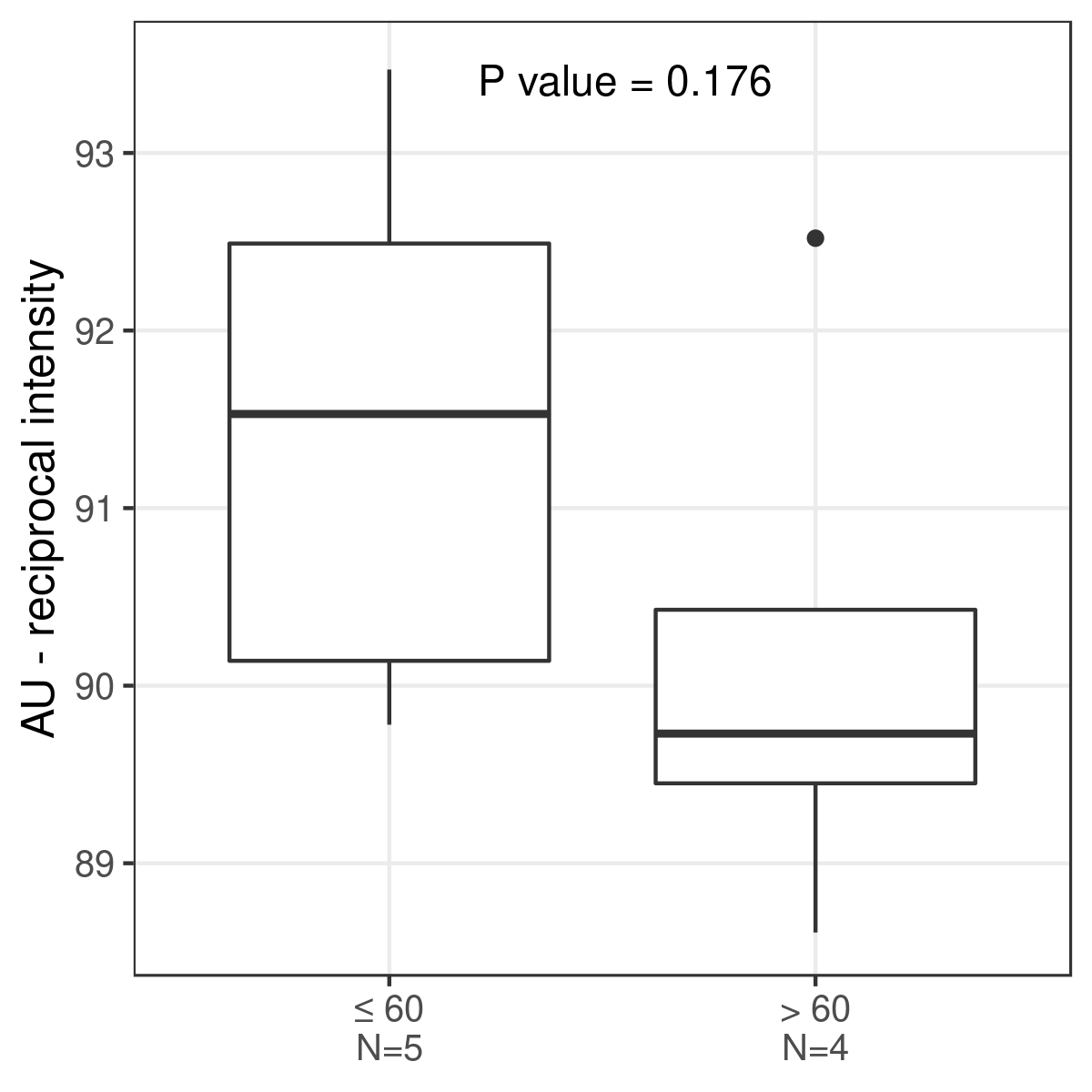


#

# Figure S4. Analysis of the difference in immunohistochemistry-derived signal intensity for kidney TSPYL5 between younger (aged 60 years or younger – <60) and older (aged over 60 years – >60) individuals from TRANSLATE Study.

N – number of individuals, P value – level of statistical significance from a Mann-Whitney U test.
