# Supplementary material for: Uncovering genetic mechanisms of kidney aging through transcriptomics, genomics, and epigenomics
Source: Kidney Int. 2019 Mar;95(3):624–35. doi: 10.1016/j.kint.2018.10.029 (PMC6390171; doi:10.1016/j.kint.2018.10.029)
Supplement: Figure S5 — Associations between age, renal expression of signature genes, and their best eSNPs in the TRANScriptome of renaL humAn TissuE (TRANSLATE) Study and The Cancer Genome Atlas (TCGA). (A) Associations between the renal expression of each gene and age in the meta-analysis of the TRANSLATE Study and TCGA. Meta P value, level of statistical significance from the meta-analysis of both studies; meta false discovery rate (FDR), the level of statistical significance after correction for multiple testing. (B) Renal expression of each gene stratified on the genotype of the best eSNP in the meta-analysis of the TRANSLATE Study and TCGA. meta P value, level of statistical significance from the meta-analysis of both studies; meta FDR, the level of statistical significance after correction for multiple testing. (C) Trajectories of age-related changes in renal expression of the 4 genes stratified on the genotype of the best respective eSNP in the meta-analysis of the TRANSLATE Study and TCGA. [file mmc6.docx]

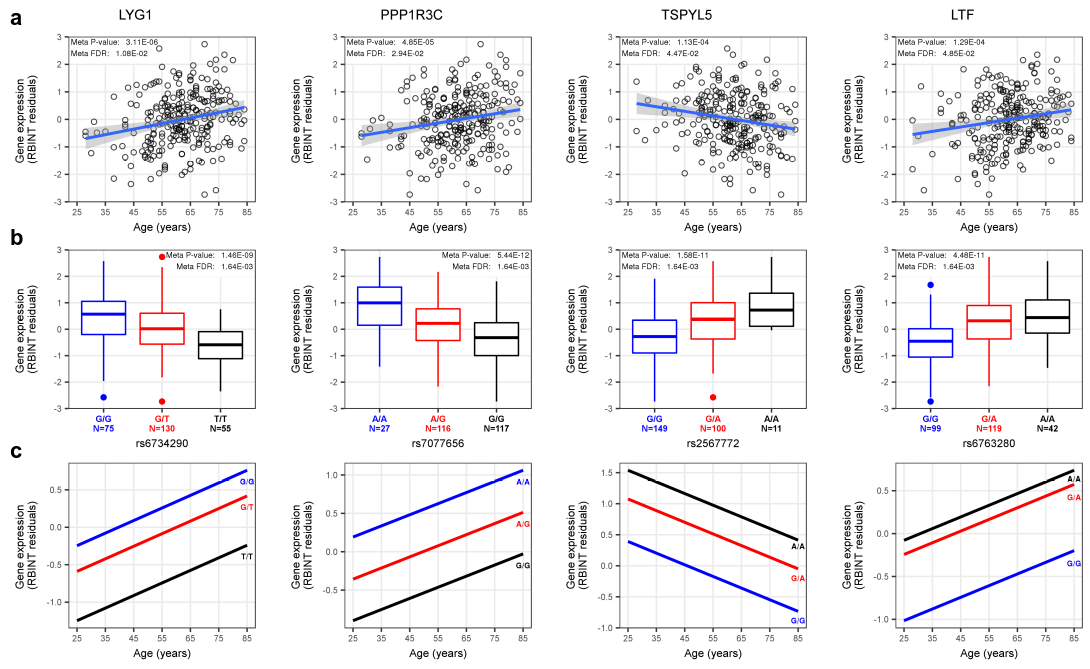


# Figure S5: Associations between age, renal expression of signature genes and their best eSNPs in TRANSLATE Study and TCGA.

**a**. Associations between the renal expression of each gene and age in the meta-analysis of TRANSLATE Study and TCGA, meta P-value – level of statistical significance from the meta-analysis of both studies, meta FDR - the level of statistical significance after the correction for multiple testing. **b**. Renal gene expression of each gene stratified on the genotype of the best eSNP in the meta-analysis of TRANSLATE Study and TCGA, meta P-value – level of statistical significance from the meta-analysis of both studies, meta FDR - the level of statistical significance after the correction for multiple testing. **c**. Trajectories of age-related changes in renal expression of the four genes stratified on the genotype of best respective eSNP in the meta-analysis of TRANSLATE Study and TCGA.
