# Supplementary material for: Uncovering genetic mechanisms of kidney aging through transcriptomics, genomics, and epigenomics
Source: Kidney Int. 2019 Mar;95(3):624–35. doi: 10.1016/j.kint.2018.10.029 (PMC6390171; doi:10.1016/j.kint.2018.10.029)
Supplement: Figure S6 — TSPYL5 functional annotation to the locus on chromosome 8. Hi-C chromatin interactions are shown as gray arcs, the intensity of gray is determined by the number of times the interaction was observed. The best mSNP and eSNP are shown as large points in blue and yellow, respectively. mSNP and eSNP statistical proxies (r2 > 0.8 in 1000 Genomes European individuals) are shown as smaller points in blue and yellow, respectively. The CpG site (cg22328208) is shown in dark purple and its parent CpG island (chromosome 8: 98289605-98290404; 25% CpG content) is shown in light purple. The TSPYL5 gene is shown as a gray region, with the coding exon black. Chromatin state information from adult kidney tissue is shown below the gene, red denotes transcription start site regions, yellow indicates enhancer regions, and green indicates transcribed regions. Input histone modification chromatin immunoprecipitation (ChIP)-seq data signal is shown at the bottom; H3K4me3 is shown in red, H3K4me1 is shown in yellow, and H3K36me3 in green. The histone modification signal is calculated as the Loess smoothed density of ChIP-seq reads across the region, colors are from Roadmap Epigenomics. [file mmc7.docx]

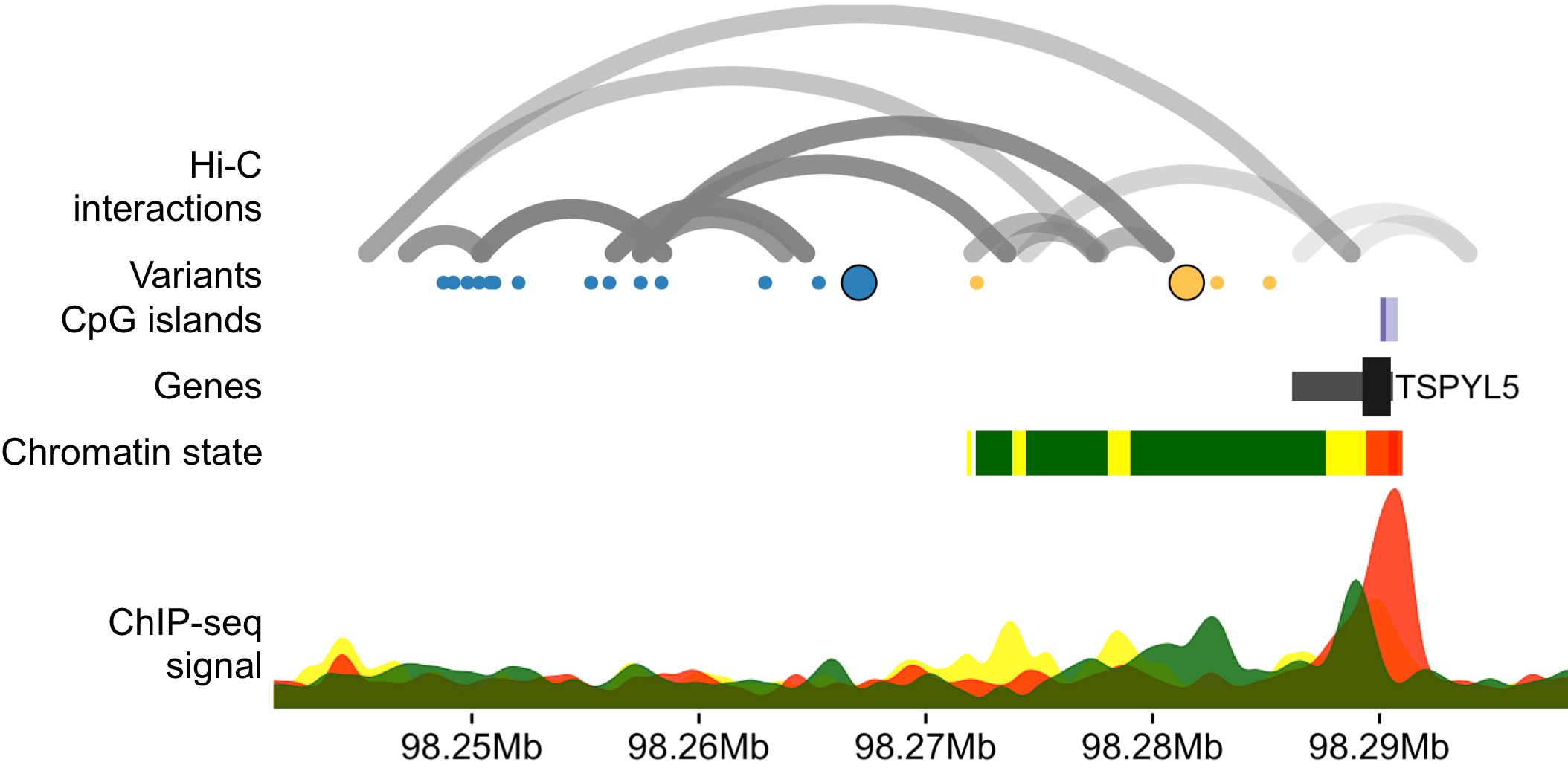


# Figure S6. TSPYL5: functional annotation to the locus on chromosome 8.

Hi-C chromatin interactions are shown as grey arcs, the intensity of grey is determined by the number of times the interaction was observed. The best mSNP and eSNP are shown as large points coloured blue and yellow, respectively. mSNP and eSNP statistical proxies (r^2^>0.8 in 1000 Genomes European individuals) are shown as smaller points coloured blue and yellow, respectively. The CpG site (cg22328208) is shown in dark purple and its parent CpG island (chr8:98289605-98290404, 25% CpG content) is shown in light purple. The *TSPYL5* gene is shown as a grey region, with the coding exon black. Chromatin state information from adult kidney tissue is shown below the gene, red denotes transcription start site regions, yellow - enhancer regions and green - transcribed regions. Input histone modification ChIP-seq data signal is shown at the bottom; H3K4me3 is shown in red, H3K4me1 is shown in yellow and H3K36me3 in green. Histone modification signal is calculated as the Loess smoothed density of ChIP-seq reads across the region, colours are from Roadmap Epigenomics.
